# Cholesterol biosynthetic pathway induces cellular senescence through ERRα

**DOI:** 10.1038/s41514-023-00128-y

**Published:** 2024-01-12

**Authors:** Dorian V. Ziegler, Joanna Czarnecka-Herok, Mathieu Vernier, Charlotte Scholtes, Clara Camprubi, Anda Huna, Amélie Massemin, Audrey Griveau, Christelle Machon, Jérôme Guitton, Jennifer Rieusset, Arnaud M. Vigneron, Vincent Giguère, Nadine Martin, David Bernard

**Affiliations:** 1 Centre de Recherche en Cancérologie de Lyon, Inserm U1052, CNRS UMR 5286, Centre Léon Bérard, Université de Lyon, Lyon, France; 2 Equipe Labellisée la Ligue Contre le Cancer, Lyon, France; 3 Goodman Cancer Research Centre, McGill University, Quebec Montreal, Canada; 4 Biochemistry and Pharmacology-Toxicology Laboratory, Lyon-Sud Hospital, Hospices Civils de Lyon, F-69495 Pierre Bénite, France; 5 CarMeN Laboratory, INSERM U1060, INRA U1397 Lyon, France; 6 Departments of Biochemistry, Medicine and Oncology, McGill University, Montreal, Quebec Montreal, Canada

**Keywords:** Senescence, Pathogenesis, Ageing

## Abstract

Cellular senescence is a cell program induced by various stresses that leads to a stable proliferation arrest and to a senescence-associated secretory phenotype. Accumulation of senescent cells during age-related diseases participates in these pathologies and regulates healthy lifespan. Recent evidences point out a global dysregulated intracellular metabolism associated to senescence phenotype. Nonetheless, the functional contribution of metabolic homeostasis in regulating senescence is barely understood. In this work, we describe how the mevalonate pathway, an anabolic pathway leading to the endogenous biosynthesis of poly-isoprenoids, such as cholesterol, acts as a positive regulator of cellular senescence in normal human cells. Mechanistically, this mevalonate pathway-induced senescence is partly mediated by the downstream cholesterol biosynthetic pathway. This pathway promotes the transcriptional activity of ERRα that could lead to dysfunctional mitochondria, ROS production, DNA damage and a p53-dependent senescence. Supporting the relevance of these observations, increase of senescence in liver due to a high-fat diet regimen is abrogated in ERRα knockout mouse. Overall, this work unravels the role of cholesterol biosynthesis or level in the induction of an ERRα-dependent mitochondrial program leading to cellular senescence and related pathological alterations.

## Introduction

Cellular senescence is a program promoted by a myriad of stresses such as telomeres shortening, oncogenic stress-induced hyper-replication and oxidative stress-mediated damages^1^. This program is characterized by a permanent cell cycle arrest and a senescence-associated secretory phenotype (SASP)^1^, both implicated in the pathophysiological effects of senescent cells^2,3^. Senescence-associated pathophysiological contexts include development, tissue regeneration, cancer and aging^2,3^. Although timely regulated senescence exerts beneficial effects, the accumulation of senescent cells throughout life and upon exposure to chronic stresses exerts detrimental effects by promoting aging and its associated diseases^4–9^. Mechanistically, while downstream factors and effectors, such as p53, p21^CIP1^ and p16^INK4A^ or NF-κB and C/EBPβ, respectively blocking cell cycle progression or promoting SASP, were extensively studied^1–3^, upstream molecular and subcellular mechanisms controlling these factors are less understood.

Senescent cells harbour metabolic changes related to both catabolism and anabolism^1,10–15^, to the point that some metabolic specificities of senescent cells are used to target or detect them^11,16,17^. Indeed, senescent cells display metabolic rearrangements as evidenced for instance by an altered glycolytic state and glucose utilization^11,12,18^, a deregulated mitochondrial metabolism, an AMPK activation and an altered NAD^+^ metabolism^10,19–22^. Lipid metabolism is also modified in senescent cells^15,23–30^. For instance, senescent hepatocytes^26,27^, fibroblasts^31^, and T-cells^32^ display an accumulation of lipid droplets (LD), this later accounting mostly for an increase of free fatty acids^23^ and free cholesterol^25,28^, subsequently esterified and incorporated in LD via triglycerides (TG) or cholesteryl esters. The mevalonate (MVA) pathway is part of lipid anabolism and involved in the endogenous biosynthesis of poly-isoprenoids, such as prenyl groups, ubiquinone, cholesterol or dolichol^33^. MVA pathway is thus crucial for many cellular processes including protein-protein interactions, mitochondrial respiration, membranes fluidity or glycosylation^33^. Some studies using pharmacological tools, such as statins or bisphosphonates, have previously suggested an involvement of this pathway in regulating senescence, still with some contradictory effects reported^34,35^. Furthermore, how endogenous cholesterol biosynthesis mechanistically regulate cellular senescence remains so far elusive. Interestingly, in a functional genetic screen of a constitutively active kinase library that we previously reported^36^, two kinases of the MVA pathway, mevalonate kinase (MVK) and phosphomevalonate kinase (PMVK), were identified as able to induce premature senescence when ectopically expressed. Still the mechanisms of action of these enzymes in this context and the relevance of these observations during senescence in vivo remained unknown.

In this study, we assessed through the use of genetic tools whether and how the MVA pathway and the downstream biomolecules regulate cellular senescence. Unexpectedly, this work led to the identification of a new mechanism regulating senescence: a cholesterol-dependent ERRα transcriptional program leading to mitochondrial dysregulation and senescence-associated liver alterations.

## Results

### Mevalonate pathway promotes cellular senescence

**Fig. 1:**
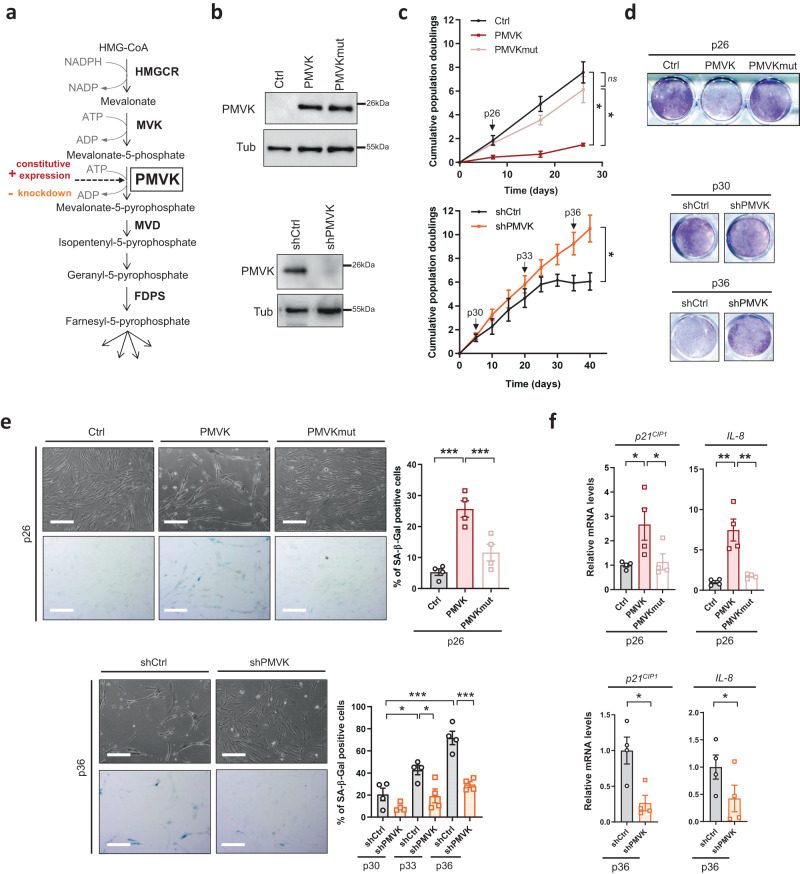
The MVA pathway regulates cellular senescence in normal cells. **a** Schematic representation of the MVA pathway in eukaryotic cells. HMGCR, HMG-CoA reductase; MVK, mevalonate kinase; PMVK, phosphomevalonate kinase; MVD, mevalonate-diphosphate decarboxylase; FDPS, farnesyl diphosphate synthase. Constitutive expression (in red) or knockdown by shRNA (in orange) was performed in MRC5 fibroblasts. **b** Relative PMVK and Tubulin protein levels in empty vector (Ctrl), PMVK- or Kinase Dead (PMVKmut) PMVK-expressing cells and in shCtrl- or shPMVK-expressing cells. **c** Growth curves of Ctrl, PMVK- or PMVKmut-expressing cells, and shCtrl and shPMVK-expressing cells. Mean +/− SEM of *n* = 4 independent biological replicates. RM one-way ANOVA test on last time point. **d** Crystal violet staining of Ctrl, PMVK- or PMVKmut-expressing cells at early passage (p26), and of shCtrl- and shPMVK-expressing cells at early (p30) and late (p36) passage. **e** Micrographs and quantification of SA-β-gal positive cells of Ctrl, PMVK- or PMVKmut-expressing cells at early passage (p26), and of shCtrl- and shPMVK-expressing cells during passages. Mean +/− SEM of *n* = 4 independent biological replicates. Scale bar: 100 µm. RM one-way ANOVA test (upper panel) and paired Student’s T-test (lower panel). **f** RT-qPCR of *p21*^*CIP1*^ and *IL-8* genes in Ctrl, PMVK- or PMVKmut-expressing cells at early passage (p26), and of shCtrl- and shPMVK-expressing cells at late passage (p36). Mean +/− SEM of *n* = 4 independent biological replicates. RM one-way ANOVA test (upper panel) and paired Student’s T-test (lower panel). ns (nonsignificant; *p < 0.05; **p < 0.01; ***p < 0.001).

In order to investigate whether the MVA pathway can regulate senescence, we expressed in MRC5 normal human fibroblasts a constitutively active PMVK^37^ and its K22M mutant, a mutant reported to have lost its kinase activity^38^, to examine whether the kinase activity of PMVK is required to induce premature senescence (Fig. 1a, b and Supplementary Fig. 1A, B). We confirmed that K22M mutant did not display any kinase activity showing that it was indeed a PMVK kinase-dead mutant (PMVKmut) (Supplementary Fig. 1C). We also expressed an shRNA directed against PMVK (shPMVK) to examine the role of the MVA pathway in two relevant pro-senescence contexts, namely replicative senescence that occurs when replicative limit has been reached and progerin-induced premature senescence when compared to expression of non-mutated lamin A (Fig. 1a, b and Supplementary Fig. 1A, B). Progerin, a protein driving Hutchinson-Gilford progeria syndrome (HGPS), is encoded by the *LMNA* mutated gene. Progerin corresponds to a truncated prelamin A that is unable to be processed in a mature lamin A perturbing nuclear envelope and cell homeostasis and leading to premature senescence^39^. Remarkably, the constitutive overexpression of PMVK, but not the kinase-dead mutant PMVK, led to decreased cell proliferation (Fig. 1c, d). Most importantly, the knockdown of PMVK was sufficient to extend the replicative potential of normal human fibroblasts over successive passages (Fig. 1c, d) and expressing progerin, when compared to lamin A (Supplementary Fig. 1D, E). The overexpression of PMVK, but not its kinase-dead version, was also associated with the induction of key markers of cellular senescence: senescence-associated β-galactosidase (SA-β-gal) activity (Fig. 1e) and increased mRNA levels of *p21*^*CIP1*^ and SASP marker *IL-8* (Fig. 1f). On the contrary, decreasing PMVK reduced SA-β-gal activity and decreased mRNA levels of *p21*^*CIP1*^ and *IL-8* in normal fibroblasts at late passages (p36) (Fig. 1e, f) and expressing progerin (Supplementary Fig. 1F, G). Together these results show that PMVK contributes to replicative and progerin-induced senescence and its kinase activity participates in senescence regulation.

To further prove that this observed senescence is mediated by the MVA pathway and not the sole PMVK enzyme, we stably expressed a shRNA against PMVK and subsequently expressed the upstream enzyme MVK (Fig. 1a). As expected, constitutive overexpression of MVK (Supplementary Fig. 1H) induced premature cellular senescence, as shown by decreased cell density (Supplementary Fig. 1I, J), elevated SA-β-gal activity (Supplementary Fig. 1K) and induction of both *p21*^*CIP1*^ and *IL-8* mRNA levels (Supplementary Fig. 1L). Noteworthy, the knockdown of PMVK abolished MVK-induced premature senescence, rescuing decreased cell density (Supplementary Fig. 1I, J), and increased SA-β-gal activity and *p21*^*CIP1*^ and *IL-8* mRNA levels (Supplementary Fig. 1K, L).

Taken together, these data support that the MVA pathway plays a pivotal role in promoting cellular senescence.

### Dysfunctional mitochondria, ROS production, DNA damage and p53 mediate mevalonate pathway-induced senescence

**Fig. 2:**
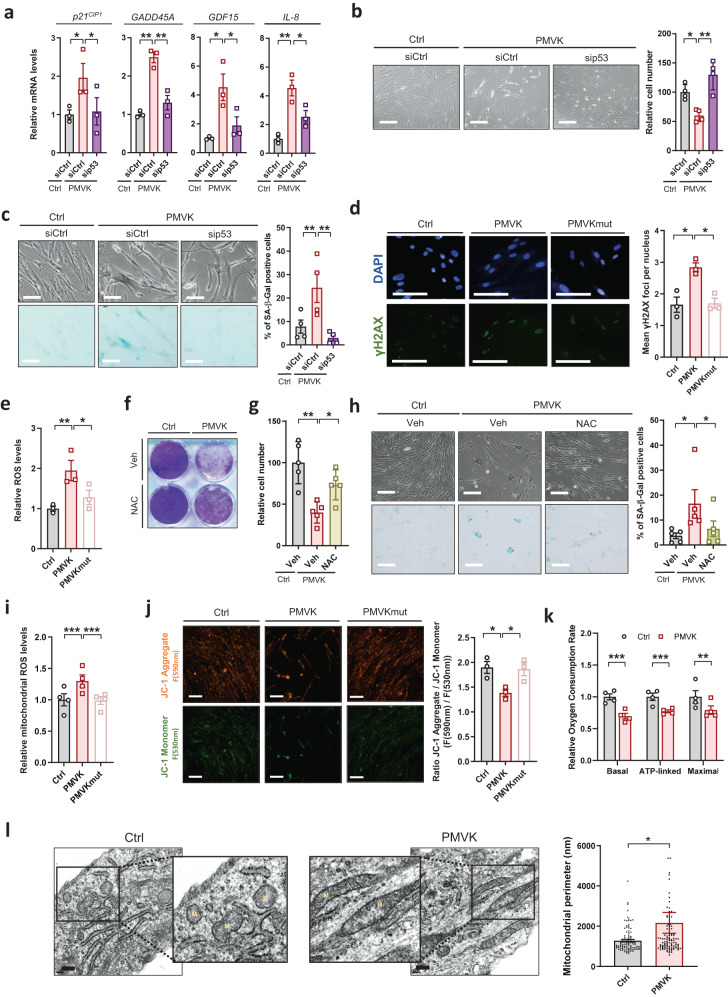
MVA pathway induction promotes mitochondrial dysfunction, ROS accumulation, DNA damage and p53-dependent senescence. **a** RT-qPCR of p53-regulated genes, including *p21*^*CIP1*^, *GADD45A*, *GDF15* and *IL-8* in Ctrl or PMVK-expressing cells previously transfected with control non-targeting siRNA (siCtrl) or p53-targeted siRNA (sip53). Mean +/− SEM of *n* = 3 independent biological replicates. RM one-way ANOVA test. **b** Micrographs of Ctrl and PMVK-expressing cells upon sip53. Quantification of relative cell number in Ctrl and PMVK-expressing cells. Mean +/− SEM of *n* = 4 independent biological replicates. Scale bar: 100 µm. RM one-way ANOVA test. **c** Representative micrographs and quantification of SA-β-gal positive cells in Ctrl and PMVK-expressing cells upon siCtrl or sip53. Mean +/− SEM of *n* = 4 independent biological replicates. Scale bar: 50 µm. RM one-way ANOVA test. **d** Micrographs of Ctrl, PMVK- or PMVKmut-expressing cells immunostained with γH2AX antibody and DAPI stained. Scale bar: 10 µm. Number of γH2AX foci were counted manually using FiJi and divided by the number of nuclei to evaluate the mean number of foci per nucleus. Fully stained nuclei were excluded from this analysis. Mean +/− SEM of *n* = 3 independent biological replicates. RM one-way ANOVA test. **e** Quantification of relative ROS levels using CellROX™ Green Reagent probe and measuring its fluorescence intensity in Ctrl, PMVK- and PMVKmut- expressing cells. Mean +/− SEM of *n* = 3 independent biological replicates. RM one-way ANOVA test. **f** Crystal violet staining after 8 days in Ctrl and PMVK-expressing cells upon vehicle (Veh) or N-acetyl-cysteine (NAC) antioxidant treatment. **g** Cell count in Ctrl and PMVK-expressing cells upon vehicle or NAC treatment. Mean +/− SEM of *n* = 5 independent biological replicates. RM one-way ANOVA test. **h** Representative micrographs and quantification of SA-β-gal positive cells in Ctrl and PMVK-expressing cells upon the vehicle or NAC treatment. Mean +/− SEM of *n* = 5 biological replicates. Scale bar: 10 µm. RM one-way ANOVA test. **i** Quantification of mitochondrial ROS using mitochondrial hydroxyl radical detector and measuring its fluorescence intensity in Ctrl, PMVK-, and PMVKmut-expressing cells. Mean +/− SEM of *n* = 4 independent biological replicates. RM one-way ANOVA test. **j** Mitochondrial membrane polarisation measurement assessed by JC1-probe. Representative micrographs of JC1 in both monomer (F(530 nm)-green) or aggregate (F(590 nm)-orange) forms within Ctrl, PMVK-, and PMVKmut-expressing cells. Scale bar: 100 µm. Quantification of ratio fluorescence intensity (Monomer) / fluorescence intensity (Aggregate). Mean +/− SEM of *n* = 3 independent biological replicates. RM one-way ANOVA test. **k** Seahorse quantitative analysis of relative oxygen consumption rate (OCR). Basal OCR was evaluated in resting conditions, ATP-linked OCR upon Oligomycin treatment and Maximal OCR upon FCCP treatment. Mean +/− SEM of *n* = 5 independent biological replicates. Multiple paired Student’s T-tests. **l** Representative electron micrographs of mitochondria in Ctrl and PMVK-expressing cells and quantification of mitochondrial perimeters. M: Mitochondria. Mean +/− SD of *n* = 103 (Ctrl) and *n* = 106 (PMVK) mitochondria. Representative of *n* = 3 independent biological experiments. Scale bar: 300 nm. (*p < 0.05; **p < 0.01; ***p < 0.001).

P53 is a well-known critical effector of cellular senescence^40^. Its transcriptional targets such as *p21*^*CIP1*^, *GADD45A* and *GDF15* were upregulated at mRNA levels by the constitutive expression of PMVK (Fig. 2a) and their up-regulations were p53-dependent as they were prevented by p53 knockdown using siRNA (Fig. 2a), suggesting that p53 could mediate PMVK-induced senescence. Indeed, p53 knockdown in PMVK overexpressing cells (Supplementary Fig. 2A) reverted several features of senescence such as PMVK-induced p53 targets and *IL-8* mRNA expression (Fig. 2a), decrease in cell number (Fig. 2b) and increase in SA-β-Gal activity (Fig. 2c).

During cellular senescence, p53 activation can occur through oxidative stress-induced DNA damage^1^ and its subsequent DNA damage response^2,3^. Accordingly, p53 pathway activation upon PMVK constitutive expression resulted from increased oxidative stress - DNA damage pathway as cells overexpressing PMVK displayed increased DNA damage evidenced by increased γH2AX positive cells (Fig. 2d), concomitantly with increased total ROS levels (Fig. 2e). Our results support that ROS is upstream of p53 as siRNA against p53 did not decrease ROS production (Supplementary Fig. 2B) whereas it decreased senescence (Fig. 2a–c) upon PMVK overexpression. Of note, cells overexpressing the kinase-dead mutant PMVK displayed a limited increase of γH2AX positive cells and an absence of ROS generation (Fig. 2d, e). Importantly, NAC antioxidant treatment largely overcame PMVK-induced senescence (Fig. 2f–h) showing a critical role of ROS in mediating PMVK-induced senescence. In the context of replicative senescence, the sole knockdown of PMVK was sufficient to reduce γH2AX positive cells and ROS levels (Supplementary Fig. 2C, D).

Mitochondria is one of the major sites of ROS production in the cell^41^ and its dysregulation participates in cellular senescence^13,19,42^. Strikingly, constitutive overexpression of PMVK, but not of kinase-dead mutant PMVK, led to increased mitochondrial ROS generation as evidenced by enhanced intensity of specific mitochondrial ROS probe (Fig. 2i). In order to evaluate mitochondrial dysfunction in PMVK-expressing cells, we assessed mitochondrial membrane potential using cationic JC-1 dye^43^, but also mitochondrial oxygen consumption rate (OCR) through Seahorse mitochondrial stress test (Supplementary Fig. 2E)^44^. PMVK-induced cellular senescence is accompanied by a drop of mitochondrial membrane potential (Fig. 2j), which could be promoted by ROS production and/or increase ROS production, and a concomitant decrease of mitochondrial ETC functions, as evidenced by reduced mitochondrial basal, ATP-linked and maximal respiration (Fig. 2k and Supplementary Fig. 2E). Dysfunctional mitochondria may harbor changes in their morphology^45,46^. Related to mitochondrial dysfunction observed in PMVK-expressing cells, we also identified atypical and persistent enlarged morphology of mitochondria in PMVK-expressing cells using electron transmission micrographs (Fig. 2l).

All these results support that PMVK could promote senescence by dysregulating ETC mitochondrial functions, further inducing ROS production, DNA damage and p53 activation.

### The cholesterol biosynthetic branch participates in mevalonate pathway-induced senescence

**Fig. 3:**
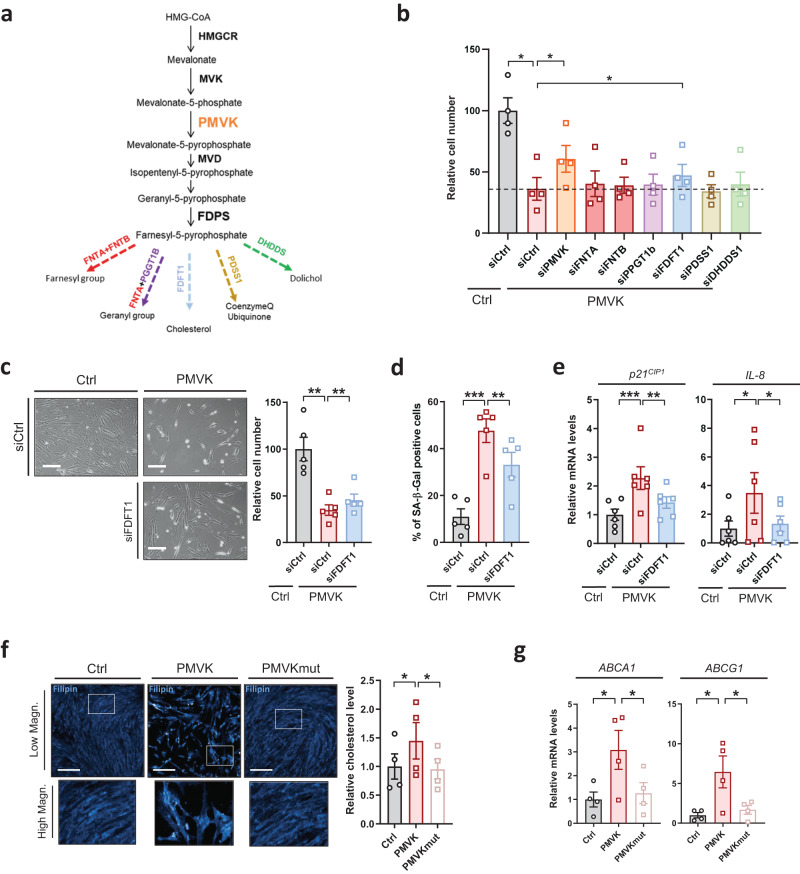
Cholesterol biosynthetic branch participates in PMVK-induced senescence. **a** Schematic representation of the MVA pathway and subbranches stemming from Farnesyl-5-Pyrophosphate, including farnesylation (red), geranylation (purple), cholesterol synthesis (blue), ubiquinone synthesis (gold) and dolichol biosynthesis (green). First specific enzymes of each subbranch are indicated. **b** Quantification of number of cells after transfection with siRNA against PMVK and enzymes of downstream subbranches of the MVA pathway in PMVK-expressing cells. Mean +/− SEM representative of *n* = 4 independent biological replicates. RM one-way ANOVA test. **c** Representative micrographs of Ctrl and PMVK-expressing cells upon siCtrl or siFDFT1 transfection (scale bar: 100 µm) and cell number quantification. Mean +/− SEM of *n* = 5 independent biological replicates. RM one-way ANOVA test). **d** Quantification of SA-β-gal positive cells in Ctrl- and PMVK-expressing cells upon siCtrl or siFDFT1 transfection. Mean +/− SEM of *n* = 5 independent biological replicates. RM one-way ANOVA test. **e** RT-qPCR of *p21*^*CIP1*^ and *IL-8* genes in Ctrl and PMVK-expressing cells upon siCtrl or siFDFT1 transfection. Mean +/− SEM of *n* = 6 independent biological replicates. RM one-way ANOVA test. **f** Cholesterol assay using filipin fluorescent sensor in Ctrl, PMVK-, and PMVKmut-expressing cells. Scale bar: 150 µm. Relative quantification of intracellular cholesterol level. Mean +/− SEM of *n* = 4 independent biological replicates. RM one-way ANOVA test. **g** RT-qPCR against *ABCA1* and *ABCG1* genes in Ctrl, PMVK-, and PMVKmut-expressing cells. Mean +/− SEM of *n* = 3 independent biological replicates. RM one-way ANOVA test. (*p < 0.05; **p < 0.01; ***p < 0.001).

Farnesyl-5-pyrophosphate is the end-product of the MVA pathway and presents three isoprene units, elementary units further used either to be transferred as prenyl groups to proteins or to be condensated into more complex poly-isoprenoids, that include among others dolichol, ubiquinone or cholesterol^33^ (Fig. 3a). In order to dissect the role of downstream branches in the PMVK-induced senescence, we knocked down the first enzyme of each branch, subsequently overexpressed PMVK and automatically counted the number of cells 4 days later. Only the knockdown of FDFT1, the first enzyme of the cholesterol biosynthesis branch, partially reverted the decreased cell number induced by PMVK overexpression (Fig. 3a, b). SiRNA-mediated FDFT1 knockdown (Supplementary Fig. 3A) also rescued PMVK-induced decrease in cell density (Fig. 3c), increase in SA-β-gal activity (Fig. 3d), and increase in *p21*^*CIP1*^ and *IL-8* mRNA levels (Fig. 3e), without impacting *PMVK* mRNA level (Supplementary Fig. 3A). Confirming these results, stable knockdown of FDFT1 by two independent shRNA partly reverted premature senescence induced by PMVK expression (Supplementary Fig. 3B–D). In line with a role of the cholesterol biosynthetic branch in inducing cellular senescence, the expression of PMVK, but not the kinase-dead mutant PMVK, boosted intracellular cholesterol content, according to the fluorescence of the cholesterol sensor filipin^47^ (Fig. 3f), and increased cholesterol-dependent LXR transcriptional targets^48^, namely *ABCA1* and *ABCG1* (Fig. 3g).

Altogether these results indicate a functional role of the cholesterol biosynthesis pathway in the regulation of PMVK-induced senescence.

### Mitochondrial master regulator Estrogen-Related receptor alpha mediates mevalonate pathway-induced senescence

**Fig. 4:**
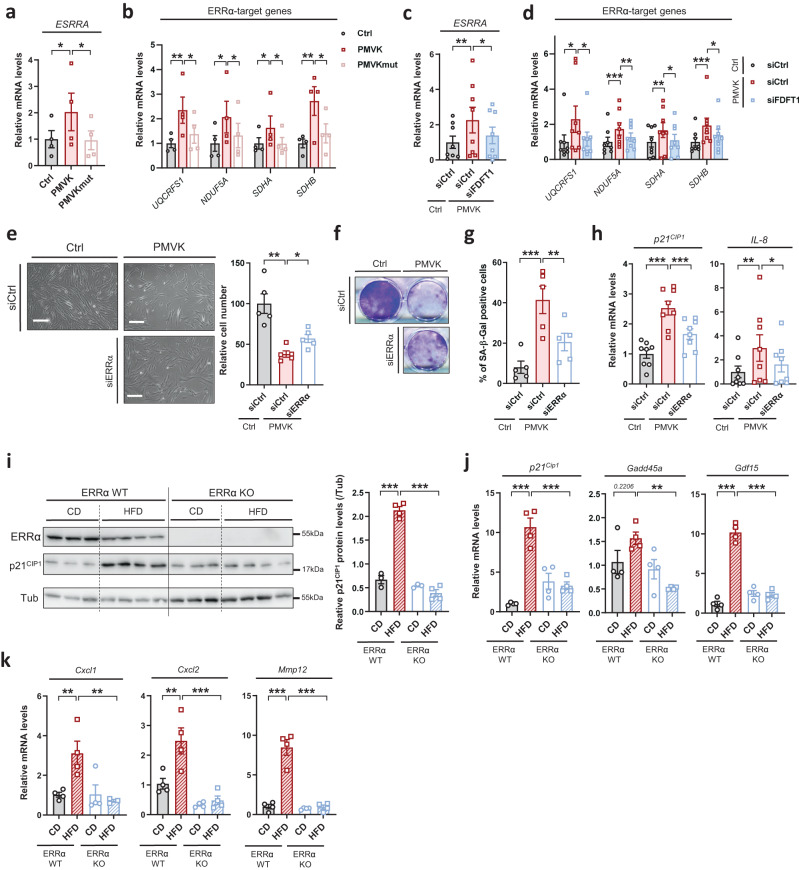
Mitochondrial master regulator Estrogen-Related Receptor alpha mediates PMVK-induced senescence. **a**, **b** RT-qPCR of *ESRRA* and ERRα target genes (including *UQCRFS1*, *NDUF5A*, *SDHA*, *SDHB*) in empty vector (Ctrl), PMVK- or Kinase Dead (PMVKmut) PMVK-expressing cells. Mean +/− SEM of *n* = 4 independent biological replicates. RM one-way ANOVA test. **c**, **d** RT-qPCR of *ESRRA* and ERRα target genes in Ctrl and PMVK-expressing cells, upon siCtrl or siERRα transfection. Mean +/− SEM of *n* = 8 independent biological replicates. RM one-way ANOVA test. **e** Representative micrographs and cell number quantification of Ctrl and PMVK-expressing cells previously transfected with siERRα. Scale bar: 100 µm. Mean +/− SEM of *n* = 5 independent biological replicates. RM one-way ANOVA test. **f** Crystal violet staining of Ctrl and PMVK-expressing cells previously transfected with siERRα. **g** Quantification of SA-β-gal positive cells in Ctrl and PMVK-expressing cells upon siERRα transfection. Mean +/− SEM of *n* = 5 independent biological replicates. RM one-way ANOVA test. **h** RT-qPCR of *p21*^*CIP1*^ and *IL-8* genes in Ctrl and PMVK-expressing cells previously transfected with siCtrl or siERRα. Mean +/− SEM of *n* = 7 independent biological replicates. RM one-way ANOVA test. **i** Western blot on ERRα, p21^CIP1^ and Tubulin in liver of ERRα WT and ERRα KO male mice fed either by chow diet (CD) or high-fat diet (HFD). Quantification of p21^CIP1^ levels normalized to Tubulin levels. Mean +/− SEM of *n* = 3–4 male mice. Ordinary one-way ANOVA test. **j**, **k** RT-qPCR of p53 target genes (namely *p21*^*Cip1*^, *Gadd45a, Gdf15*) and SASP members *Cxcl1, Cxcl2 and Mmp12* genes in liver of ERRα WT and ERRα KO male mice fed either by chow diet (CD) or high-fat diet (HFD). Mean +/− SEM of *n* = 3–4 mice. Ordinary two-way ANOVA test. (*p < 0.05; **p < 0.01; ***p < 0.001).

Estrogen-Related Receptor alpha (ERRα or NR3B1) is a key regulator of mitochondrial functions and of metabolism^49,50^. Noteworthy, mRNA levels of ERRα were upregulated in both replicative and progerin-induced senescence (Supplementary Fig. 4A). We then assessed during PMVK-induced senescence the expression of ERRα targets: ERRα itself^51^ encoded by *ESRRA* gene and *UQCRFS1*, *NDUF5A*, *SDHA* and *SDHB* nuclear-encoded mitochondrial targets^49^. The constitutive overexpression of PMVK increased the expression of *ESRRA*, *UQCRFS1*, *NDUF5A*, *SDHA* and *SDHB* (Fig. 4a, b and Supplementary Fig. 4B, C). Remarkably, this ERRα program upregulation was partly reverted by the knockdown of FDFT1 (Fig. 4c, d and Supplementary Fig. 4B–D) suggesting the hypothesis of ERRα activation by the cholesterol biosynthetic pathway during PMVK-induced senescence. Further confirming an activation of ERRα during PMVK-induced senescence, its knockdown by siRNA strategy blocked PMVK-induced upregulation of various ERRα targets (Supplementary Fig. 4E, F), without impacting PMVK levels (Supplementary Fig. 4G).

We next sought to determine whether cholesterol-dependent ERRα program functionally mediates the PMVK-induced senescence. Knockdown of ERRα in PMVK-overexpressing cells partially rescued the decreased cell number (Fig. 4e, f), the increased SA-β-gal activity (Fig. 4g), and the elevated *p21*^*CIP1*^ and *IL-8* mRNA levels (Fig. 4h). Further supporting that ERRα mediates MVA/cholesterol biosynthetic pathway-induced senescence, its chemical inhibition using XCT-790 compound, similarly to ERRα knockdown, largely bypassed senescence induced by PMVK expression according to growth curves and SA-β-Gal activity (Supplementary Fig. 4H, I).

To next investigate the relevance of these discoveries in a relevant pathophysiological system, we investigated the level of cellular senescence in the liver of ERRα knockout mice fed by high-fat diet (HFD), known to increase cholesterol levels and drive cellular senescence and senescence-dependent steatosis^27,52^. ERRα target genes *Uqcrfs1*, *Nduf5a*, *Sdha* and *Sdhb* were only slightly induced by the HFD, potentially because some endogenous ERRα activity upon the chow diet and were accordingly reduced at mRNA levels in the liver of ERRα knockout mice, especially upon HFD (Supplementary Fig. 4J). As expected, HFD resulted in the accumulation of senescence markers such as *p21*^*Cip1*^ (Fig. 4i, j), *Gadd45a* and *Gdf15* p53 targets (Fig. 4j), *Mmp12* SASP factor (Fig. 4k), and pro-inflammatory SASP factors *Cxcl1* and *Cxcl2* (Fig. 4k), which are murine functional orthologs of human *IL8*^53^. Strikingly, increase in the expression of these senescence markers was abrogated in liver of ERRα knockout mice upon HFD (Fig. 4i–k). As previously reported^54^, HFD-fed ERRα knockout mice did not display hepatic steatosis, a process tightly linked to senescent cell accumulation^27,52^. Examination of the same senescence markers in white adipose tissue (WAT), which can display increased senescence during HFD^55^, displayed some similarities and differences with the liver. Indeed, *p21*^*Cip1*^, *Gdf15*, *Mmp12*, and *Cxcl2* were induced whereas *Gadd45a and Cxcl1* were not in the control mice by the HFD (Supplementary Fig. 4K). Induction by the HFD of *Gdf15*, *Mmp12*, *Cxcl2* was decreased in the ERRα knockout mice (Supplementary Fig. 4K).

Overall, these results highlight the importance of ERRα in mediating MVA/cholesterol biosynthetic pathway-induced cellular senescence in vitro in human cells and in vivo in mouse.

## Discussion

**Fig. 5:**
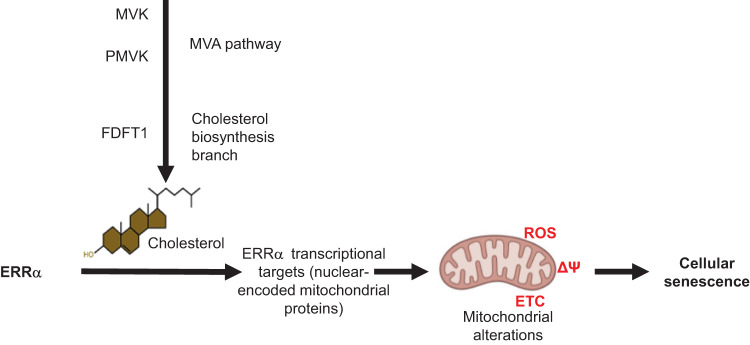
A key role for cholesterol-ERRα axis in promoting cellular senescence. The experimental models, human cells through manipulating the MVA pathway or WT or ERRα KO mice subjected or not to a HFD (high-fat diet), indicate that cholesterol accumulation triggers ERRα activation. In both in vitro and in vivo conditions, ERRα is determinant to mediate the subsequent cellular senescence phenotype. Mechanistically demonstrated in vitro, the upregulation of ERRα target genes enhances mitochondrial dysfunction, through electron transport chain (ETC) alteration, drop in mitochondrial membrane potential (ΔΨ) and ROS production, ultimately resulting in cellular senescence.

In this study, we deciphered the role of the MVA pathway, the cholesterol biosynthetic pathway and of ERRα transcription factor in mediating cellular senescence. MVA pathway activation triggers premature senescence whereas its inhibition delays replicative and progerin-induced senescence in normal human cells. PMVK-induced senescence seems to mediate, at least partly, by the biosynthetic cholesterol pathway and the activation of an ERRα transcriptional program, mitochondrial ROS accumulation, DNA damage and p53 activation. ERRα is also mediating cellular senescence in the liver, and to a lower extent in the WAT, when mice are subjected to a high fat diet (Fig. 5).

Several studies depicted a role of MVA pathway in cellular senescence through the use of statins and aminobisphosphonates inhibitors but with conflicting conclusions. For instance blocking the MVA pathway with these inhibitors has been shown to delay senescence in HUVEC model^34^, whereas it promotes senescence in oral keratinocytes and has no obvious effect in oral fibroblasts^35^. Besides, statins and aminobiphosphonates have been shown both to blunt SASP, notably pro-inflammatory cytokines including IL-6, IL-8 and monocyte chemoattractant protein (MCP)-1^56–59^, and some of its effects, including pro-tumorigenic activity^60^. Dampening SASP through statins could explain their numerous anti-inflammatory outcomes in vivo^61,62^. Our results using genetic tools, knockdown and/or overexpression of PMVK or MVK, clearly demonstrate that the MVA pathway promotes cellular senescence and associated SASP in normal human fibroblasts. Discrepancies with pharmacological studies might thus result from the known pleiotropic effects of the MVA inhibitors, as these inhibitors were found to exert multiple MVA pathway-independent actions^63,64^. For instance, bisphosphonates in addition to inhibiting FDPS enzyme and downstream pathway also induce accumulation of the upstream metabolite isopentenyl diphosphate (IPP) which can induce apoptosis, at least in some specialized cells^57^.

Although the MVA pathway contributes to senescence as its knockdown delays replicative and progerin-induced senescence, how its activity is regulated during senescence remains unknown. PMVK is up-regulated during progerin-induced senescence but not during replicative senescence, with the same trends for FDFT1, whereas MVK levels are not modified (Supplementary Fig. 5A, B). Nonetheless, cholesterol levels are increased during both replicative or progerin-induced senescence (Supplementary Fig. 5C, D). Taken together, these results suggest an accumulation of cholesterol in senescent cells, in line with two recent studies displaying this accumulation in other senescence models^65,66^, and that this accumulation is not directly due to an up-regulation of PMVK and FDFT1. We can then speculate that post-translational modifications, as previously reported with two upstream MVA enzymes, namely HMGCR phosphorylation^67^ or HGMCS sumoylation^68^, are involved in regulating the MVA pathway during senescence. Last but not least, cholesterol level is regulated not only by synthesis but also through transport and/or degradation^69^. Thus, the exact contribution of MVA pathway in the accumulation of cholesterol in these senescence models would further require deeper investigations.

Our results suggest an unexpected link between the MVA pathway and the DNA damage/p53 pathway, a well-known effector pathway promoting cellular senescence^70^. This DNA damage/p53 pathway could be activated by an oxidative stress upon PMVK-induced senescence, probably mainly provoked by increased mitochondrial ROS production. Mitochondrial ROS are well known mediators of cellular senescence and can result from multiple mitochondrial alterations, from increased respiration and thus increased basal electron leaks during the electron transport chain activity, to decreased respiration but increased levels of electron leaks because of altered electron transport chain complex associations^41^. According to our findings, PMVK-induced senescence results in decreased respiration with increased mitochondrial ROS production. This is in line with previous results showing that maladaptive complex 1 assembly through increased transcriptional program of ETC components results in decreased respiration with increased mitochondrial ROS and promotes cellular senescence and aging^71^.

Our siRNA approach to reveal which sub-branch(es) of the MVA pathway participate in PMVK-induced senescence identified an involvement of the cholesterol biosynthetic branch. Of note, this finding does not exclude that some other sub-branches could collaborate with the cholesterol biosynthetic pathway to regulate cellular senescence. Beyond the specific role of ceramide^24^, little is known about links between lipids and senescence except that the lipid metabolism is largely modified in senescent cells. In particular and in accordance with our results in replicative and progerin-induced senescence, it has been reported that free fatty acids and cholesterol levels are increased in senescent cells^25,26,28,29^ and that ceramides or prostaglandins regulate also the onset of senescence^24,72^. In line with our findings, recent articles reported an upregulation of the expression of cholesterol synthesis genes in a model of oncogene-induced senescence^73^ and an increased cholesterol during DNA damage-induced senescence^66^. During OIS, knockdown of some cholesterol synthesis genes partially bypassed cell proliferation arrest. However, the mechanisms behind these observations were not deciphered^73^. During DNA damage-induced senescence, cholesterol is thought to promote the SASP by increasing mTORC1 activity^66^. Our results underline the importance of cholesterol biosynthetic branch downstream of the MVA pathway in the regulation of cellular senescence and allow us to propose a mechanistic model through ERRα activation. Our findings and the findings of others do not exclude additional mechanisms, dependent or not on ERRα, controlled by the cholesterol in cellular senescence, including in a paracrine manner, as it has been described for some other lipids^74^.

Our results support that ERRα participates in the pro-senescence function of the cholesterol biosynthetic pathway as its knockdown or its chemical inhibition partially overcomes PMVK-induced premature senescence. Although ablation of FDFT1, ERRα or p53 decrease PMVK-induced senescence, their effects show slight differences and not complete reversal of the phenotype suggesting that other factors and pathways could also contribute to PMVK-induced senescence. A previous study proposed that cholesterol can be an ERRα ligand^50^, as this is still largely debated further investigation will be needed to confirm or not this observation. As far as we know ERRα has not been associated with cellular senescence. Nevertheless, it has been shown to be activated during cancer cell death in response to PLA2R1/JAK/STAT signalling, this signalling being known to promote cellular senescence in normal human cells and in mice^58,75–77^. In cancer cells, PLA2R1/JAK/STAT signalling in an ERRα-dependent manner blocks mitochondrial respiration, alters mitochondria and mediates ROS generation^78^, reminiscent to the observed alterations during PMVK-induced senescence in normal cell.

We have previously reported that ERRα-null mice are protected from HFD-induced non-alcoholic fatty liver disease (NAFLD)^54^, which is characterized by liver steatosis. Senescent cells accumulate in the liver during HFD and/or aging and contribute to liver steatosis^27,52^. We observed that ERRα-depleted livers are protected from increased senescence, suggesting that reduced ERRα-induced senescence could contribute to the decreased steatosis in the liver of these mice^54^. Adding another layer of complexity, body weight (BW) is decreased in ERRα KO mice upon HFD^54^, suggesting that BW decrease could contribute to decrease senescence and steatosis observed in the liver of these mice. As far as we know whether senescent cell accumulation can impact BW is currently unknown. Further studies will thus be needed to better define the complex functional connection between BW, senescence and steatosis. Still, mechanistically, it has been previously shown that ERRα loss^54^ or elimination of senescent cells^27^ results in change in free fatty acid metabolism decreasing liver steatosis. Beyond these observations highlighting the importance of ERRα in regulating senescence and senescence-dependent liver steatosis, we can speculate that other known pathophysiological processes regulated by ERRα could rely on its effect on cellular senescence. For instance, our results in WAT also support a decreased cellular senescence in the ERRα-null mice. ERRα-null mice display reduced osteoporosis in female mice^79,80^ and cellular senescence in the bone is known to promote osteoporosis^81^, suggesting that ERRα-promoted senescence could participate in ERRα-induced osteoporosis. In the same line, antiosteoporosis effects of MVA pathway inhibitors, aminobisphosphonates, are well-known^82^ and may partly rely on reduced ERRα activity, though further studies need to critically test this hypothesis. Effect of ERRα in vivo on senescence can then be wide in term of tissue physiology impacted and then still needs to be deeply investigated in the future. For instance, ERRα is expressed in different cell types in the mouse liver, according to publicly available single-cell RNA sequencing, raising the question of the cells impacted by ERRα on senescence occurrence during HFD. In the context of ERRα-driven cellular senescence in vivo, it will also be critical to characterize mitochondrial phenotype in tissue as our results support it is a critical mediator of MVA pathway/ERRα-induced senescence.

Overall, our results emphasize MVA pathway/cholesterol biosynthetic pathway, cholesterol levels and subsequent ERRα program as a new pathway regulating cellular senescence. As impacting cellular senescence has attracted many interests to improve various age-related diseases and health span^7–9^, our work paves the way to new potential senotherapeutic strategies using drugs targeting the mevalonate pathway (statins, bisphosphonates) and in particular PMVK, and ERRα.

## Methods

### Cell culture and reagents

MRC5 normal human fibroblasts (ATCC, Manassas, VA, USA), and kidney 293 GP cells (Clontech, Mountain View, CA, USA) were cultured in Dulbecco′s modified Eagle′s medium (DMEM, Life Technologies, Carlsbad, USA) with GlutaMax and supplemented with 10% FBS (Sigma-Aldrich, Saint-Louis, USA) and 1% penicillin/streptomycin (ThermoFisher Scientific). XCT-790 (Medchem, HY-10426/CS-2413) was used at 150 nM. N-Acetyl-Cysteine (NAC) (A9165, Sigma-Aldrich) was used directly after infection at 1 mM. These compounds were renewed every two days.

### Vectors, transfection and infection

Retroviral vectors were used to constitutively overexpress MVK, PMVK, lamin A and progerin. MVK and PMVK plasmids were provided by Addgene in the Myristoylated Kinase Library (Kit #1000000012) described in^37^. K22M mutation of the kinase-dead PMVK mutant (PMVKmut) was generated using QuickChange Site-Directed Mutagenesis Kit (Agilent, Catalog # 200518). pBABE‐puro‐GFP‐wt‐lamin A (Addgene #17662) and pBABE‐puro‐GFP‐progerin (Addgene #17663) were described in^83^. Lentiviral particles were used to constitutively express shPMVK (pLV[shRNA]-Hygro-U6> hPMVK[shRNA#4], Target sequence: GAGAACCTGATAGAATTTATC) and shFDFT1 (pLV[shRNA]-Hygro-U6 > hFDFT1[shRNA#2], Target sequence CAACGATCTC CCTTGAGTTTA and pLV[shRNA]-Hygro-U6 > hFDFT1[shRNA#3], Target sequence: ACC ATTTGAATGTTCGTAATA) and were provided by VectorBuilder. 293 T or 293GP virus-producing cells were transfected using the GeneJuice reagent according to the manufacturer’s recommendations (Merck Millipore). Two days after transfection, viral supernatant was collected, diluted with fresh medium (1/2) and hexadimethrine bromide was added (final concentration 8 μg/mL; Sigma-Aldrich). Target cells were then infected, centrifugated with virus particles for 30 min at 2000 rpm and subsequently incubated during 6 h at 37 °C 5% CO_2_. Fresh medium was added after 6 h incubation. One day later, infected cells were selected with Geneticin at 75 µg/mL, Hygromycin at 15 µg/mL (ThermoFisher Scientific), or puromycin at 500 ng/ml (Invivogen).

### siRNA

MRC5 fibroblasts were plated and reverse transfected with ON-TARGET plus SMART pool of small interference (si) RNAs: siCtrl (Catalog: D-001810-10-20 / Lot#2693147), siPMVK (Catalog: L-006782-00), siFNTA (Catalog: L-008807-00), siFNTB (Catalog: L-010093-00), siPGGT1B (Catalog: L-008703-00), siFDFT1 (Catalog: #L-009442-00-0005 / Lot#180518), siPDSS1 (Catalog: L-008464-01), siDHDDS (L-010399-01), siESRRA (Catalog: #L-003403-00-0005 / Lot#191211), sip53 (Catalog: #L-003329-00-0001) / Lot#180912) (Horizon Discovery) previously incubated for 20 min with Dharmafect 1 Transfection Reagent (Horizon Discovery) 0,6% in antibiotics- and serum-free medium DMEM with Glutamax. Final siRNA concentration in the well was 15 nM. The day after, cells were infected (with retroviral particles containing PMVK) as referenced previously.

### Animals

Wild-type (WT) and ERRα^-/-^ male mice in a C57BL/6 J genetic background were housed and fed ad libitum with free access to water in an animal facility at McGill University. All animal experiments were conducted in accord with accepted standards of humane animal care and all protocols were approved by the McGill Facility Animal Care Committee and the Canadian Council on Animal Care. For high-fat diet (HFD) experiments, mice were separated randomly into groups of two to three mice per cage and fed either a control chow diet consisting of 10 kcal percent fat (catalog no. TD.08806; Harlan, Indianapolis, IN) or an HFD consisting of 60 kcal percent fat (catalog no. TD.06414; Harlan) and known to increase cholesterol levels according to manufacturer, during 15 weeks, initiated at 6 weeks of age. For all mouse experiments, littermates were used and mice were euthanized by cervical dislocation at Zeitgeber time (ZT) 4 for tissue isolations.

### RNA extraction, reverse transcription, and real-time quantitative PCR

Total RNAs were extracted with phenol-chloroform using Upzol (Dutscher, Brumath, France). Synthesis of cDNA was performed using Maxima First cDNA Synthesis Kit (ThermoFisher Scientific) from 1 μg of total RNA. cDNA (50 ng/µL) was used as a template for quantitative PCR (qPCR), and mixed with primers (200 nM), SYBR™ Green PCR Master Mix (ThermoFisher Scientific) or TaqMan mix (Roche) and Universal Probe Library probes (100 µM) (ThermoFisher Scientific) for the gene of interest. Reactions were performed in triplicate. qPCR analyses were carried out with the FX96 Thermocycler (Biorad, Hercules, USA). Relative mRNA levels were calculated using the Comparative Ct (ΔΔCT) method. Gene expression was normalized with *hACTB* or *mRplp*. Primer sequences used are listed in Supplementary Table 1.

### Senescence-associated β-Galactosidase analysis and Crystal violet

For SA-β-Galactosidase assay, cells were washed with PBS 1X, fixed for 5 min in 2% formaldehyde / 0.2% glutaraldehyde, rinsed twice in PBS 1X, and incubated at 37 °C overnight in SA-β-Galactosidase staining solution as previously described^84^. For crystal violet staining, cells were washed with PBS 1X, fixed for 15 min in 3.7% formaldehyde and stained with crystal violet.

### ROS and JC1 quantification

Total cellular and specific mitochondrial ROS were measured respectively with CellROX™ Green Reagent (ThermoFisher Scientific) and Cell Meter™ Mitochondrial Hydroxyl Radical Detection Kit (ATT Bioquest) according to manufacturer’s recommendations. For JC1, JC1-Mitochondrial Membrane Potential Assay Kit (ab113850, Abcam) was used. JC1 monomers and aggregates were both excited at 488 nm. Detection of fluorescence for JC1 monomers and aggregates were performed respectively at 530 nm and 590 nm. Ratio F(aggregate)/F(monomer) was subsequently evaluated. Pictures acquisition was performed using Operetta CLS High-Content Analysis System (PerkinElmer). All the quantifications of ROS and JC1 were performed using Columbus Software.

### Cholesterol measurement

Relative intracellular cholesterol concentration was measured using Cholesterol Assay Kit, based on filipin fluorescent sensor^47^ (Cell-Based) (Abcam, ab133116) according to manufacturer’s recommendations. Quantification was performed using ImageJ Software.

### PMVK kinase assay

The assay is based on an enzymatic coupling system using the Pyruvate kinase (PK), Pyruvate Oxidase (PO) and the Horse Radish Peroxidase (HRP). The reaction synthetizing Diphospho-mevalonate by the PMVK is measured through the ADP produced simultaneously, detected through its capacity to engage enzymatic coupling of PK, PO, and HRP through pyruvate and H_2_O_2_ intermediates and the oxidation of Amplex red into the fluorescent compounds Resazurin. Six days after infection, MRC5 cells were washed twice with ice-cold KH buffer (50 mM HEPES, 110 mM KOAc, pH 7.2). Then cells were transferred on ice and incubated in KHM buffer (110 mM KOAc, 20 mM HEPES, 2 mM MgOAC, pH 7.2) containing 500 µg/ml of digitonin. After 5 min the supernatants were collected on ice and frozen at -20 °C. Lysates were thawed and then mixed v/v with a buffer containing HEPES pH 7.2 30 mM, NaCl 40 mM, EGTA 2 mM, Tween 20 0.04%, MgCl_2_ 20 mM, BSA 0.2%. Thirty µL of this assay buffer was deposited in black fluorescent plate (2 replicates to test the kinase activity in presence or in absence of Phosphomevalonate (PMV) in the assay). Two buffers A and B were prepared in parallel, containing respectively 50 mM KH_2_PO_4_ pH 7, 375 µM Amplex Red (Sigma-Aldrich, Ampliflu™ Red, 90101) and KH_2_PO_4_ 50 mM pH 7, MgCl_2_ 2.5 mM, FAD 25 µM, Thiamine pyrophosphate 250 µM, Phosphoenol-pyruvate 2.5 mM (Megazyme), Horse Radish Peroxidase (5 U/mL) (Sigma-Aldrich, P8375), Pyruvate Kinase (15 U/mL) (from Bacillus stearothermophilus, Nipro), Pyruvate Oxidase (1.87 U/mL) (Sorachim-PYO-311). Also a solution of ATP (S1), or a mix of ATP and phospho-mevalonate (S2), was prepared in order to obtain 400 µM of each in the assay buffer by adding 5 µL of these solutions. At this step, 5 µL of S1 or S2 was first mixed with 40 µL of buffer B and then mixed again with 20 µL of buffer A. These mixtures containing either S1 or S2 were added to each tested sample and the plate was immediately incubated at 30 °C in fluorescent plate reader (Clariostar, BMG-Labtech). Real-time increase of fluorescence of rezasurin produced from Amplex red oxidation was followed during 45 min. At the end of the experiment, slopes of the curves were analyzed to retain the linear increase of fluorescence, a time window where sufficient amount of ADP has accumulated to maintain a constant activity of the enzymatic coupling system. Slope obtained with only ATP in the reaction was subtracted from that corresponding to the same sample analyzed in presence of both ATP and PMV, and plotted as a curve of PMVK-dependent fluorescence increase by unit of time.

### Seahorse

Cells were plated in Seahorse seeding 24-well plates prior infection. Infection was performed in the 24-well plates and cells were subsequently selected for 5 days with Geneticin (ThermoFisher Scientific) at 75 µg/mL. Using Seahorse XF Cell Mito Stress Test (Agilent, Santa Clara) as described in^44^, fibroblasts were sequentially treated with 1,5 μM oligomycin, 1,5 μM phenylhydrazone (FCCP), and 0.5 μM Rotenone and Antimycin A. After assay, cells were stained with Hoescht for 10mn before proceeding to cell number counting and subsequent normalization. Data were obtained at SFR Biosciences AniRA-ImmO Platform, ENS de Lyon. Seahorse XFe Wave Software (Agilent) was used to subsequently analyze the data.

### Electron microscopy

1:1 volume of glutaraldehyde 4% was added to the culture medium and cells were incubated 15 min at 4 °C. After discarding medium/glutaraldehyde, 1:1 volume of glutaraldehyde 4% / cacodylate 0.2 M pH 7.4 was added. After fixation in glutaraldehyde 2%, cells were washed three times for 1 hr at 4 °C, post-fixed with 2% OsO4 1 hr at 4 °C, and dehydrated with an increasing ethanol gradient. Impregnation was performed with Epon A (50%) plus Epon B (50%) plus DMP30 (1.7%). Inclusion was obtained by polymerisation at 60 °C for 72 h. Ultrathin sections (approximately 70 nm thick) were cut on a UCT (Leica) ultramicrotome, mounted on 200 mesh copper grids and contrasted with uranyl acetate and lead citrate. Acquisition of at least 100 mitochondria of 10–20 independent cells per condition was performed with a Jeol 1400JEM (Tokyo, Japan) transmission electron microscope equipped with an Orius 600 camera and Digital Micrograph at CIQLE platform (UCBL-Lyon).

### Immunoblot and Immunofluorescence

For immunoblot experiments, cells were lysed in RIPA buffer. After protein quantification using Bradford assay, 30 μg of proteins were loaded and resolved by SDS-PAGE electrophoresis and transferred to nitrocellulose membranes (Bio-Rad). Membranes were blocked with TBS-Tween / Milk 5% for 1 h and incubated at 4 °C with primary antibodies overnight. Membranes were then incubated with secondary antibody for 1 h at room temperature. Detection was performed using ECL kit (Amersham). Each image of blots derive from the same experiment and all the samples of each experiment were processed in parallel. For immunofluorescence experiments, cells were washed with PBS 1X, fixed for 15 min in 3.7% formaldehyde and permeabilized with Triton 100 × 0,1% for 10 min. Blocking was performed using PBS with 20% FBS during 30 min and cells were then incubated at 4 °C with primary antibodies overnight. Cells were incubated with secondary antibody for 1 h at room temperature, and washed before proceeding to image acquisition and analyses. Quantification was performed with ImageJ software. All primary antibodies and dilutions used are listed in Supplementary Table 2.

### Statistical analysis

Individual values represent mean ± SEM of n independent biological replicates for in vitro or n mice for in vivo, as mentioned in the figure legends. Shapiro-Wilk normality tests were applied to raw data before proceeding to any analyses. All further statistical analyses and tests are clearly indicated in figure legends. For groups not following normal distributions, nonparametric Mann-Whitney test was performed. For two groups both following normal distributions, parametric tests were two-tailed and performed as following Student’s t-test (equal variance) or Welch’s t-test (non-equal variance). For more than two groups, repeated measure (RM) (for paired data) or ordinary (for unpaired data) one-way ANOVA was used and Tukey’s multiple comparisons test (with a single pooled variance) was subsequently performed. For in vivo data, two-sided Grubb’s test was performed to find outliers, which were removed from further analysis if *p value* was below 0.05, and ordinary two-way ANOVA was used. All the statistical analyses were performed using GraphPad Prism Software 9.1.0 (*ns*: non-significant; *p < 0.05; **p < 0.01; ***p < 0.001).

### Reporting summary

Further information on research design is available in the Nature Research Reporting Summary linked to this article.

### Supplementary information

Supplementary file

Reporting Summary

## Data Availability

The data that support the findings of this study are available from the corresponding authors.
