# Supplementary material for: Cholesterol biosynthetic pathway induces cellular senescence through ERRα
Source: NPJ Aging. 2024 Jan 12;10(1):5. doi: 10.1038/s41514-023-00128-y (PMC10786911; doi:10.1038/s41514-023-00128-y)

Supplemental Figure 1, Ziegler et al

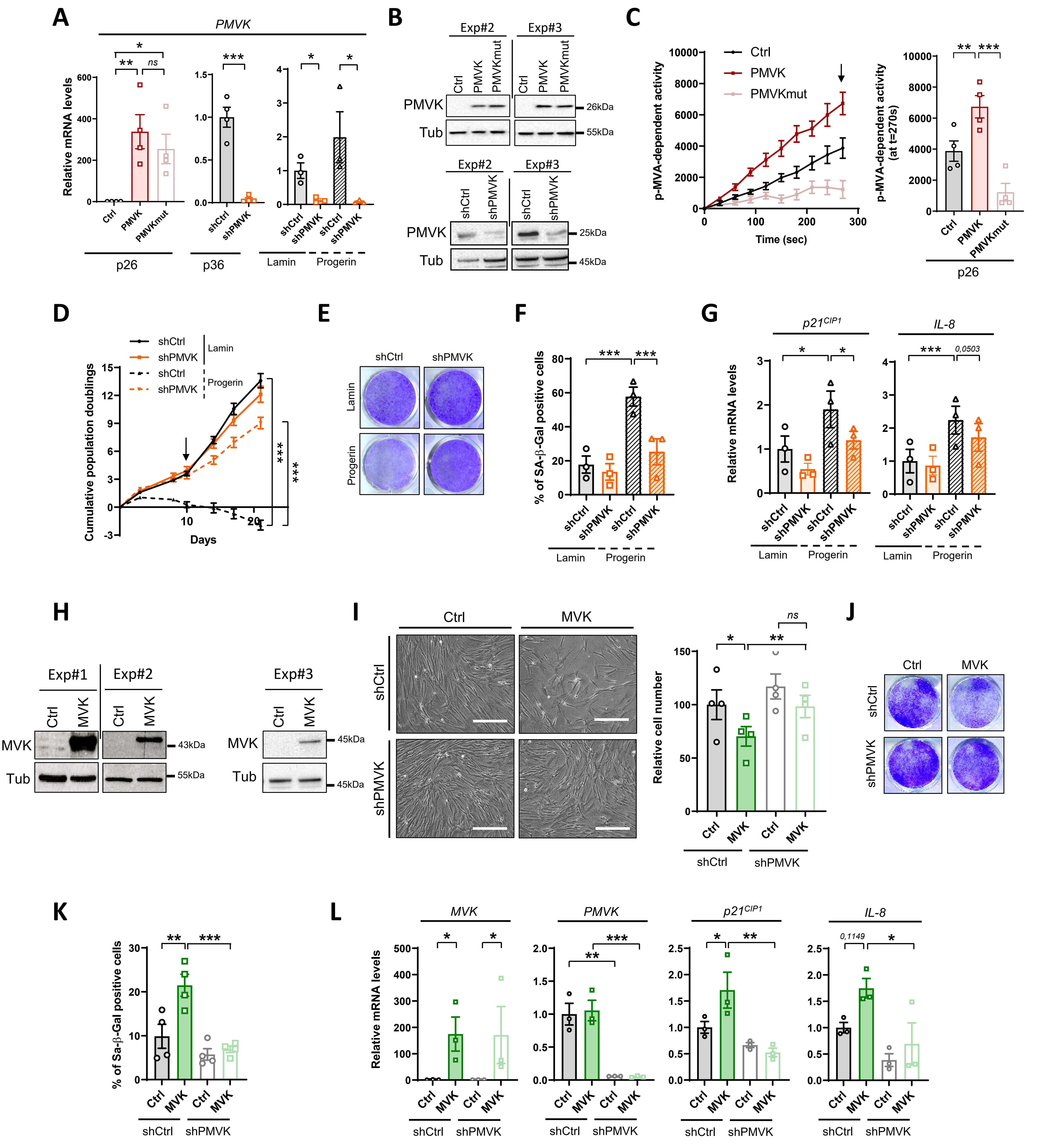

Figure 1. A. RT-qPCR of *PMVK* in Ctrl-, PMVK- or PMVKmut-expressing cells, shCtrl or shPMVK-expressing cells, and shCtrl- and shPMVK-expressing cells upon Lamin or Progerin constitutive expression. Mean +/- SEM of n=3-4 independent biological replicates. RM one-way ANOVA test and paired Student's T-test. **B.** Relative PMVK and Tubulin protein levels in empty vector (Ctrl), PMVK- or Kinase Dead (PMVKmut) PMVK-expressing cells and in shCtrl- or shPMVK-expressing cells. **C.** Phospho-Mevalonate Kinase Assay. Measurement of PMVK activity in Ctrl-, PMVK- or PMVKmut-expressing cells overtime. Mean +/- SEM of n=4 independent biological replicates. Arrow indicates end time-point at t=270sec. RM one-way ANOVA test. **D-G.** MRC5 were infected with Lamin A or progerin expressing retroviral vectors. At the end of puromycin selection, cells were seeded for different assays. **D.** Growth curves of shCtrl- and shPMVK-expressing cells upon Lamin or Progerin constitutive expression. Mean +/- SEM of n=4 independent biological replicates. RM one-way ANOVA test on last time point. Arrow indicates the time-point when D-E-F assays were performed. **E.** Crystal violet staining of shCtrl- and shPMVK-expressing cells upon Lamin or Progerin constitutive expression. **F.** Quantification of SA-β-gal positive cells of shCtrl- and shPMVK-expressing cells upon Lamin or Progerin constitutive expression. Mean +/- SEM of n=3 independent biological replicates. RM one-way ANOVA test. **G.** RT-qPCR of *p21^{CIP1}* and *IL-8* genes of shCtrl- and shPMVK-expressing cells upon Lamin or Progerin constitutive expression. Mean +/- SEM of n=3 independent biological replicates. RM one-way ANOVA test. **H.** Relative MVK and Tubulin protein levels in empty vector- (Ctrl), and MVK-expressing cells (MVK). **I.** Representative micrographs and cell number quantification after 5 days of MVK expression in shCtrl- and shPMVK-expressing cells. Scale bar: 100µm. Mean +/- SEM of n=4 independent biological replicates. RM one-way ANOVA test. **J.** Crystal violet staining after 8 days of MVK expression in shCtrl- and shPMVK-expressing cells. **K.** Quantification of SA-β-gal positive cells after 5 days of MVK expression in shCtrl- and shPMVK-expressing cells. Mean +/- SEM of n=4 independent biological replicates. RM one-way ANOVA test. **L.** RT-qPCR of *MVK*, *PMVK*, *p21^{CIP1}* and *IL-8* genes after 5 days of MVK expression in shCtrl- and shPMVK-expressing cells. Mean +/- SEM of n=3 independent biological replicates. RM one-way ANOVA test. (*ns*: non-significant; * $p < 0.05$; ** $p < 0.01$; *** $p < 0.001$).

Supplemental Figure 2, Ziegler et al

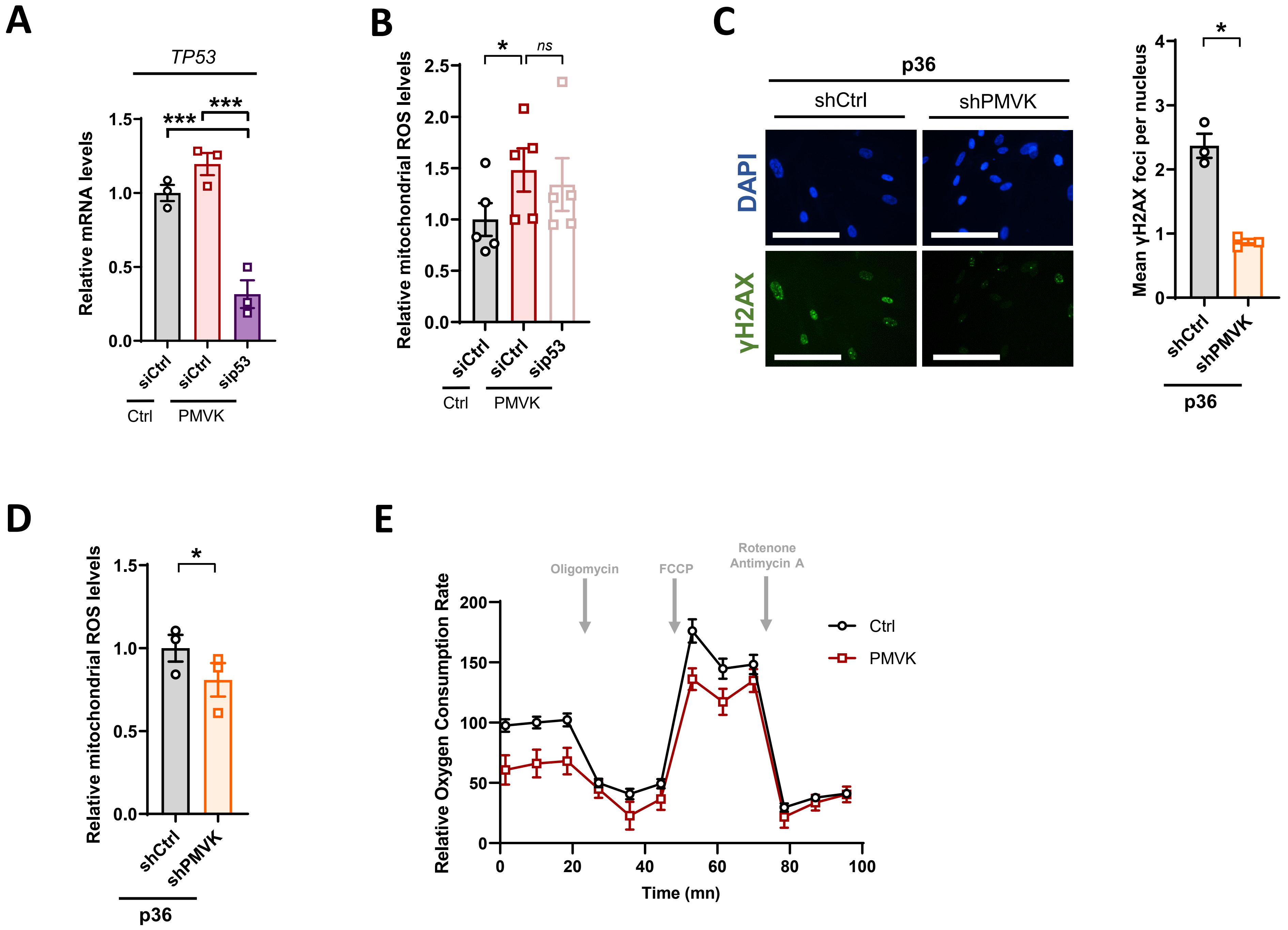

Figure 2. A. RT-qPCR of *TP53* gene in Ctrl or PMVK-expressing cells previously transfected with control non-targeting siRNA (siCtrl) or p53-targeted siRNA (sip53). Mean +/- SEM of n=3 independent biological replicates. RM one-way ANOVA test. **B.** Quantification of mitochondrial ROS in PMVK expressing cells +/- p53 siRNA. Mean +/- SEM of n=5 independent biological replicates. RM one-way ANOVA test. **C.** Micrographs of shCtrl- and shPMVK-expressing cells immunostained with γH2AX antibody and DAPI stained at late passage (p36). Scale bar: 100µm. Number of γH2AX foci were counted using FiJi (fully stained nuclei were excluded from this analysis). Mean +/- SEM of n=3 independent biological replicates. RM one-way ANOVA test. **D.** Quantification of mitochondrial ROS in shCtrl- and shPMVK-expressing cells at late passage (p36). Mean +/- SEM of n=3 independent biological replicates. RM one-way ANOVA test. **E.** Seahorse curves during mitochondrial stress test in Ctrl- or PMVK-expressing MRC5. Relative oxygen consumption rate normalized to Ctrl-MRC5. Representative curve of n=5-7 technical replicates for each condition, in a total of n=4 independent biological replicates. (*ns*: non-significant; * $p < 0.05$; ** $p < 0.01$; *** $p < 0.001$).

Supplemental Figure 3, Ziegler et al

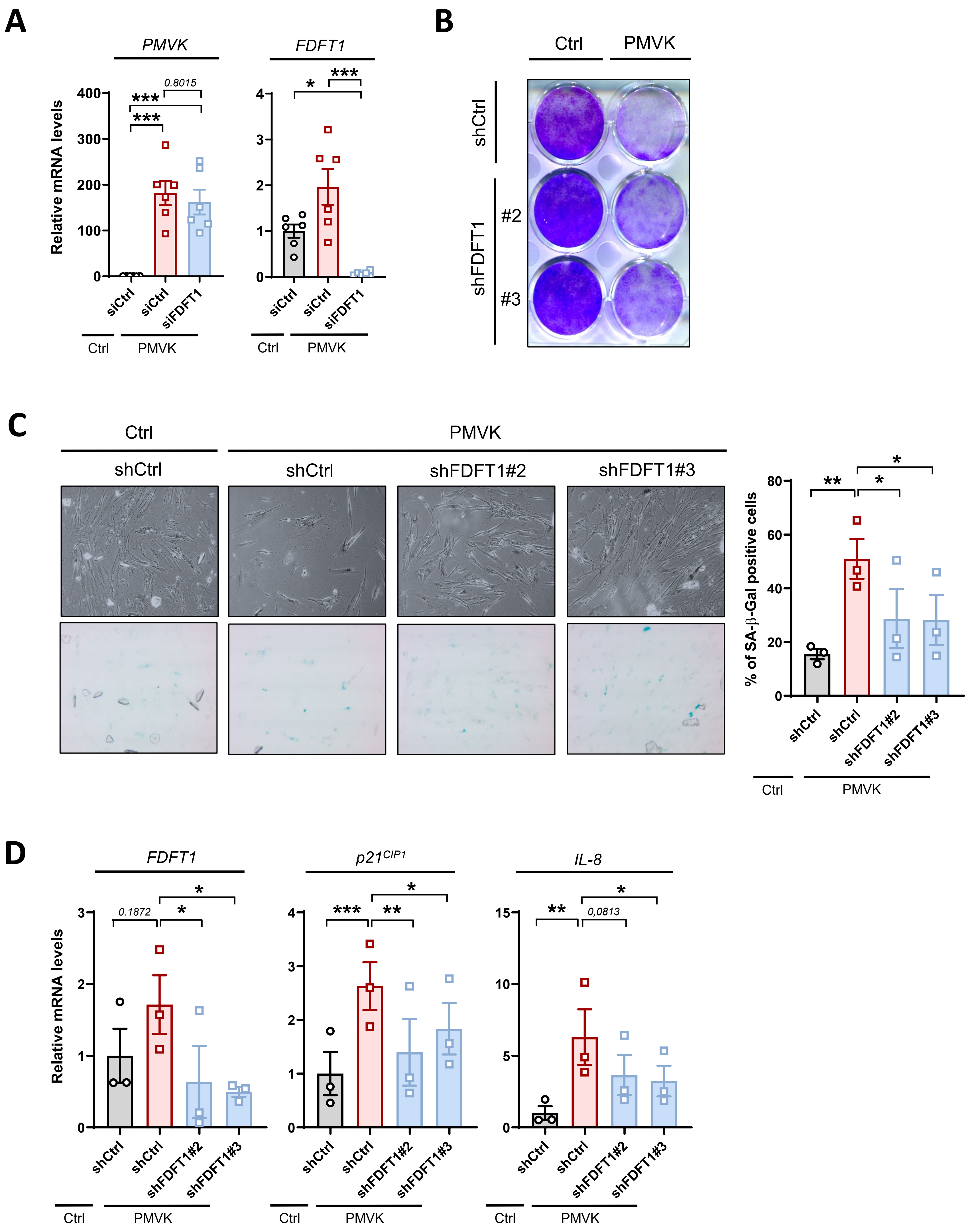

Figure 3. A. RT-qPCR of *PMVK* and *FDFT1* genes in Ctrl or PMVK-expressing cells previously transfected with control non-targeting siRNA (siCtrl) or FDFT1-targeted siRNA (siFDFT1). Mean +/- SEM of n=3-6 independent biological replicates. RM one-way ANOVA test. **B.** Crystal violet staining at day 12 after constitutive expression of PMVK in shCtrl-, shFDFT1#2- and shFDFT1#3-expressing cells. **C.** Representative micrographs and quantification of SA-β-galactosidase positive cells after constitutive expression of PMVK in shCtrl-, shFDFT1#2- and shFDFT1#3-expressing cells. Mean +/- SEM of n=3 independent biological replicates. RM one-way ANOVA test. **D.** RT-qPCR of *FDFT1*, *p21^{CIP1}* and *IL-8* genes after constitutive expression of PMVK in shCtrl or shFDFT1#2 and shFDFT1#3-expressing cells. Mean +/- SEM of n=3 independent biological replicates. RM one-way ANOVA test. (*ns*: non-significant; * $p < 0.05$; ** $p < 0.01$).

Supplemental Figure 4, Ziegler et al

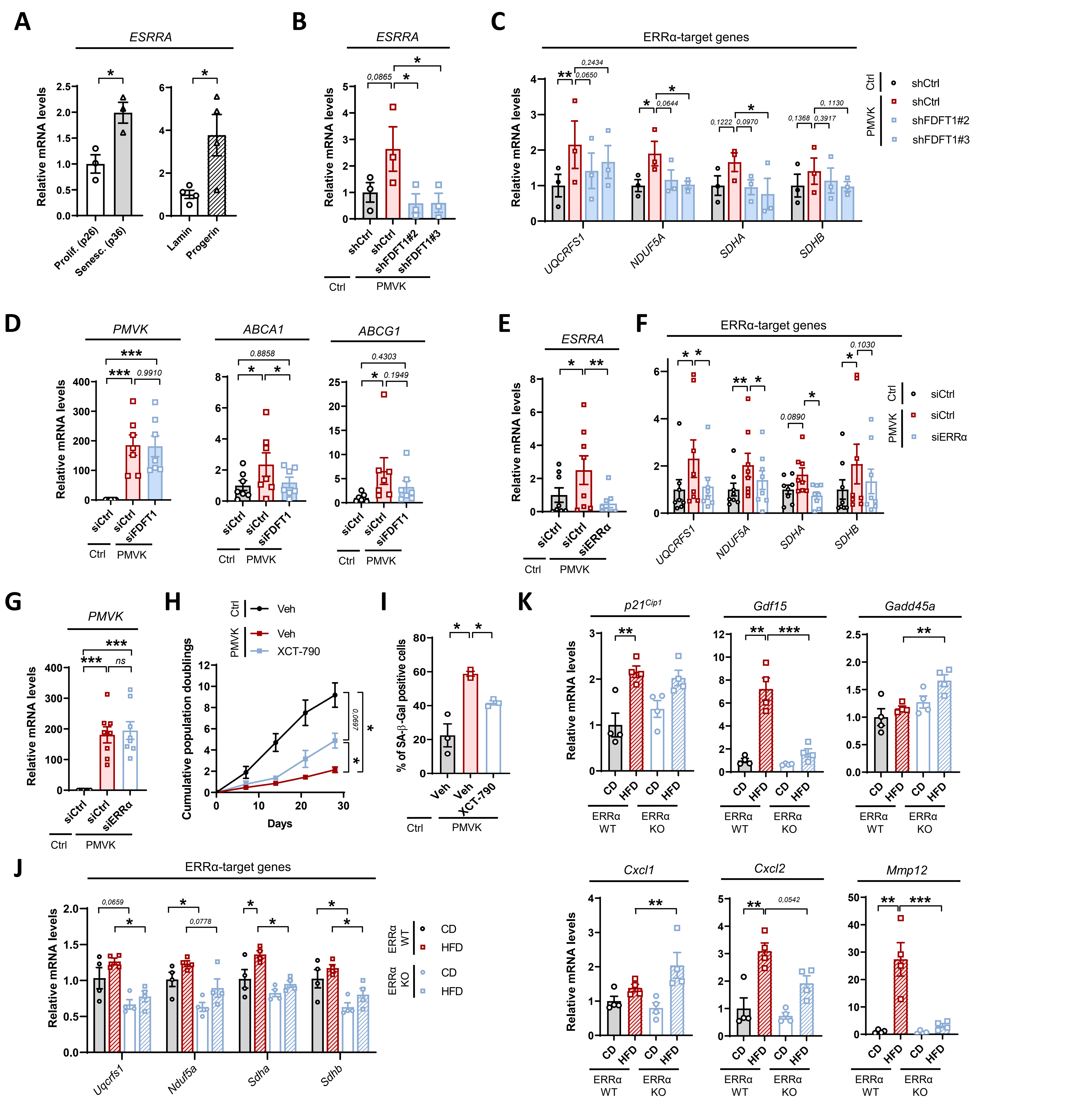

Figure 4. A. RT-qPCR of *ESRRA* in proliferating (p26) and replicative senescing (p36) or lamin- and progerin-expressing MRC5 normal cells. Mean +/- SEM of n=3-4 independent biological replicates. Unpaired Student's T-test (left panel) and paired Student's T-test (right panel). **B-C.** RT-qPCR of *ESRRA* and ERRα target genes (including *UQCRFS1*, *NDUF5A*, *SDHA*, *SDHB*) after constitutive expression of PMVK in shCtrl-, shFDFT1#2- and shFDFT1#3-expressing cells. Mean +/- SEM of n=3 independent biological replicates. RM one-way ANOVA test. **D.** RT-qPCR of *PMVK*, *ABCA1* and *ABCG1* cholesterol-dependent genes in Ctrl and PMVK-expressing cells, upon siCtrl or siERRα transfection. Mean +/- SEM of n=7 independent biological replicates. RM one-way ANOVA test. **E-F.** RT-qPCR of *ESRRA* and ERRα target genes in Ctrl and PMVK-expressing cells, upon siCtrl or siERRα transfection. Mean +/- SEM of n=8 independent biological replicates. RM one-way ANOVA test. **G.** RT-qPCR of *PMVK* gene in Ctrl and PMVK-expressing cells, either previously transfected by siCtrl or siESRRα. Mean +/- SEM of n=8 independent biological replicates. RM one-way ANOVA test. **H.** Growth curves of Ctrl and PMVK-expressing cells treated every 2 days with the ERRα inhibitor XCT-790. Mean +/-SEM of n=3 independent biological replicates. RM one-way ANOVA test on last time point. **I.** Quantification of SA-β-gal positive cells in Ctrl and PMVK-expressing cells treated or not with XCT-790. Mean +/-SEM of n=3 independent biological replicates. RM one-way ANOVA test. **J.** RT-qPCR of ERRα target genes (*Uqcrfs1*, *Ndufa5*, *Sdha*, *Sdhb*) in liver of ERRα WT and ERRα KO male mice fed either by chow diet (CD) or high-fat diet (HFD). Mean +/- SEM of n=4 mice. **K.** RT-qPCR of genes involved in senescence in WAT of ERRα WT and KO male mice fed either by chow diet (CD) or high-fat diet (HFD). Mean +/- SEM of n=4 mice. Ordinary two-way ANOVA test. (*ns*: non-significant; * $p < 0.05$; ** $p < 0.01$; *** $p < 0.001$).

Supplemental Figure 5, Ziegler et al

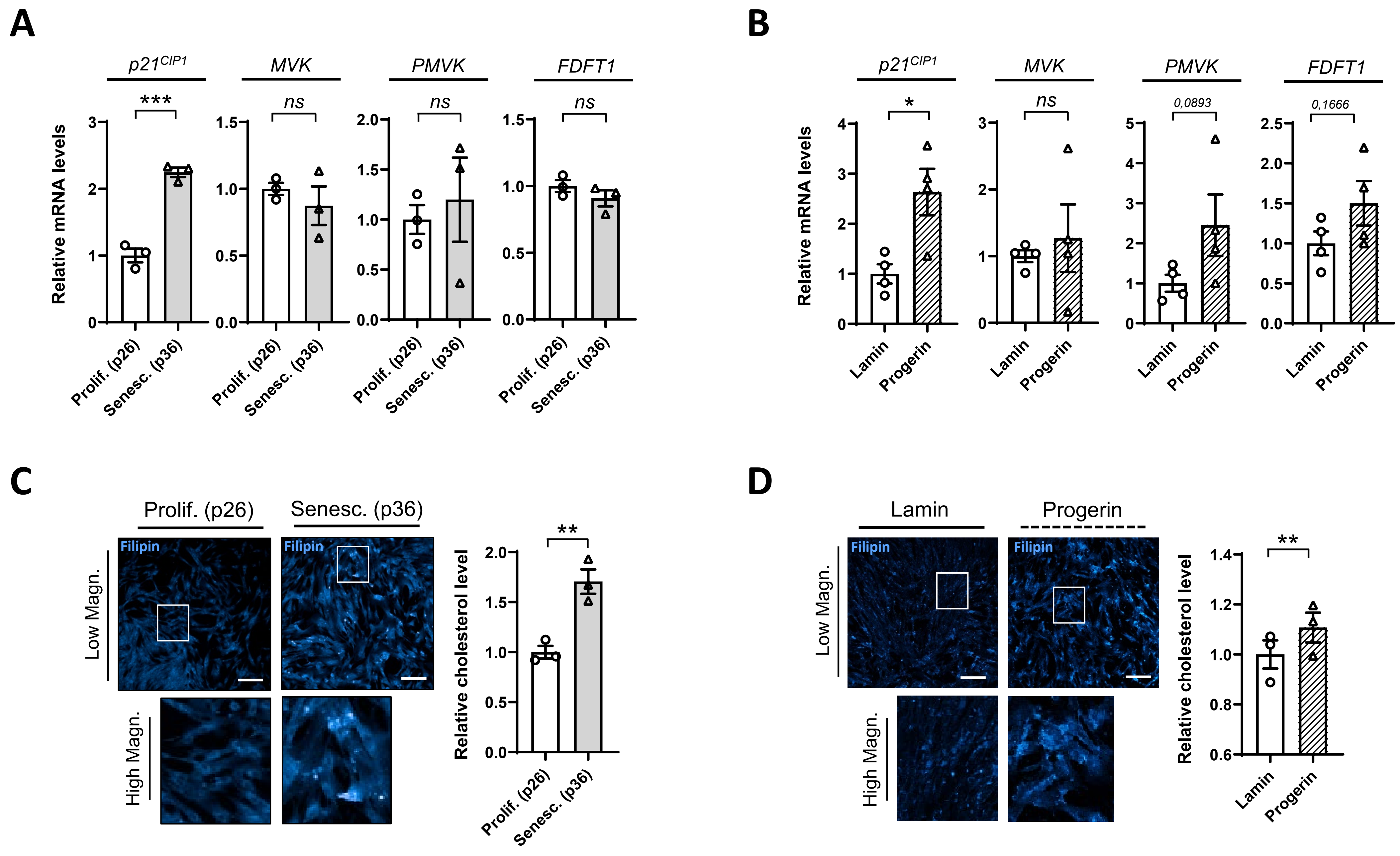

Figure 5. A. RT-qPCR of *p21*^{CIP1}, *PMVK* and *FDFT1* genes in proliferating (p26) and replicative senescing (p36) MRC5. Mean +/- SEM of n=3 independent biological replicates. Paired Student's T-test. **B.** RT-qPCR of *p21*^{CIP1}, *PMVK* and *FDFT1* genes in lamin- and progerin-expressing MRC5. Mean +/- SEM of n=4 independent biological replicates. Paired Student's T-test. **C-D.** Cholesterol assay using filipin fluorescent sensor in proliferating and senescent MRC5 (**C**) or in lamin- and progerin-expressing MRC5 (**D**). Scale bar: 150µm. Relative quantification of intracellular cholesterol level. Mean +/- SEM of n=3 independent biological replicates. RM one-way ANOVA test. (*ns*: non-significant; * $p < 0.05$; ** $p < 0.01$; *** $p < 0.001$).

Supplemental Table 1, Ziegler et al

Table 1. List of primers.

Gene	Sequences
Mouse	
Rplp-Forward	GCAGCAGATCCGCATGTCGCTCCG
Rplp-Reverse	GAGCTGGCACAGTGACCTCACACGG
p21^{CIP1}-Forward	TGAGCTGCGCCAGCTGAGGTGTG
p21^{CIP1}-Reverse	TGCCGCATGGGTTCTGACGGAC
Gadd45a-Forward	GCTCGGAGTCAGCGCACCAT
Gadd45a-Reverse	GCCACATCCCGGTCGTCGTC
Gdf15-Forward	TGCATGCCAACCAGAGCCGA
Gdf15-Reverse	GACCCTGACTCAGCGACGCC
Cxcl1-Forward	GGGGCGCCTATCGCCAATGA
Cxcl1-Reverse	AAGGCAAGCCTCGCGACCAT
Cxcl2-Forward	GAGCTTGAGTGTGACGCCCC
Cxcl2-Reverse	TCTTCCGTTGAGGGACAGCAGC
Mmp12-Forward	CTGGGCTTCTCTGCATCTGTGA
Mmp12-Reverse	AGGCTTGATTCCTGGGAAGTGT
Gadd45a-Forward	GCTCGGAGTCAGCGCACCAT
Gadd45a-Reverse	GCCACATCCCGGTCGTCGTC
Gdf15-Forward	TGCATGCCAACCAGAGCCGA
Gdf15-Reverse	GACCCTGACTCAGCGACGCC
Uqcrfs1-Forward	ATGTGAAGCGACCCTTCCT
Uqcrfs1-Reverse	ATGGGAAAAACGGACAGAAG
Nduf5a-Forward	TGGAAGAGGTGATTCTTCAGG
Nduf5a-Reverse	TATTGGCCACTTCCACTGGT
Sdha-Forward	CAGTTCCACCCCACAGGTA
Sdha-Reverse	TCTCCACGACACCCTTCTGT
Sdhb-Forward	CTGGTGGAACGGAGACAAGT
Sdhb-Reverse	GCGTTCCTCTGTGAAGTCGT
Human	
ACTB-Forward	ATTGGCAATGAGCGGTTC
ACTB-Reverse	GGATGCCACAGGACTCCAT
p21^{CIP1}-Forward	TCACTGTCTTGTACCCTTGTGC
p21^{CIP1}-Reverse	GGCGTTTGGAGTGGTAGAAAT
MVK-Forward	GCCTTTCTTTACTTATACCTGTCCA
MVK-Reverse	CCGACCACACTACGATATCCA
PMVK-Forward	TTTTGCAGGAAGATTGTGGA
PMVK-Reverse	CTCCGTGTGTCACTCACCA
IL8-Forward	AGACAGCAGAGCACACAAGC
IL8-Reverse	ATGGTTCCTTCCGGTGGT
TP53-Forward	AGGCCTTGGAACTCAAGGAT
TP53-Reverse	CCCTTTTTGGACTTCAGGTG
GADD45A-Forward	AGAGCAGAAGACCGAAAGGA
GADD45A-Reverse	TGACTCAGGGCTTTGCTGA
GDF15-Forward	CCGGATACTCACGCCAGA
GDF15-Reverse	AGAGATACGCAGGTGCAGGT
FDFT1-Forward	AGTTTCGCAGCTGTTATCCAG
FDFT1-Reverse	GATAAAATATGCACACTGCGTTG
ESSRA-Forward	GGCGGCAGAAGTACAAGC
ESSRA-Reverse	ATTCACTGGGGCTGCTGT
UQCRFS1-Forward	AGCCTGTGTTGGACCTGAAG
UQCRFS1-Reverse	TGGGAATAACAAACAGAAGCAG
NDUF5A-Forward	GGTGTGCTGAAGAAGACCACT
NDUF5A-Reverse	TTGTGTACAATATTCTTAGCCTCTCG
SDHA-Forward	TCCACTACATGACGGAGCAG
SDHA-Reverse	CCATCTTCAGTTCTGCTAAACG
SDHB-Forward	GGGGCCTGCAGTTCTTATG
SDHB-Reverse	AGGCGCTCCTCTGTGAAGT
ABCA1-Forward	CCACAAAAACATTGCTGCAT
ABCA1-Reverse	GTCACTCCAGCTTCTCATGCT
ABCG1-Forward	GTGTCGGCACATCTGAAGC
ABCG1-Reverse	AGCGCTGTCAGTATCTCCTTG

Supplemental Table 2, Ziegler et al

Protein	Reference	Use	Dilution
MVK	sc-27585 (Santa-Cruz)	WB Sup Figure 1	1/500
PMVK	sc-390775 (Santa-Cruz)	WB Figure 1 & Sup Figure 2	1/500
Tub	T6199 (Sigma-Aldrich)	WB Figure 1	1/5000
GammaH2AX	2577S (Cell Signaling)	IF Figure 2	1/300
ERRα	Ab76228 (Abcam)	WB Figure 4	1/1000
$p21^{CIP1}$	sc-817 (SantaCruz)	WB Figure 4	1/1000
Tub	CLT9002 (Cedarlane)	WB Figure 4	1/2000

Table 2. List of antibodies.

Supplemental materials, Ziegler et al

Uncropped Western blot

To generate Figure 1B – upper panel

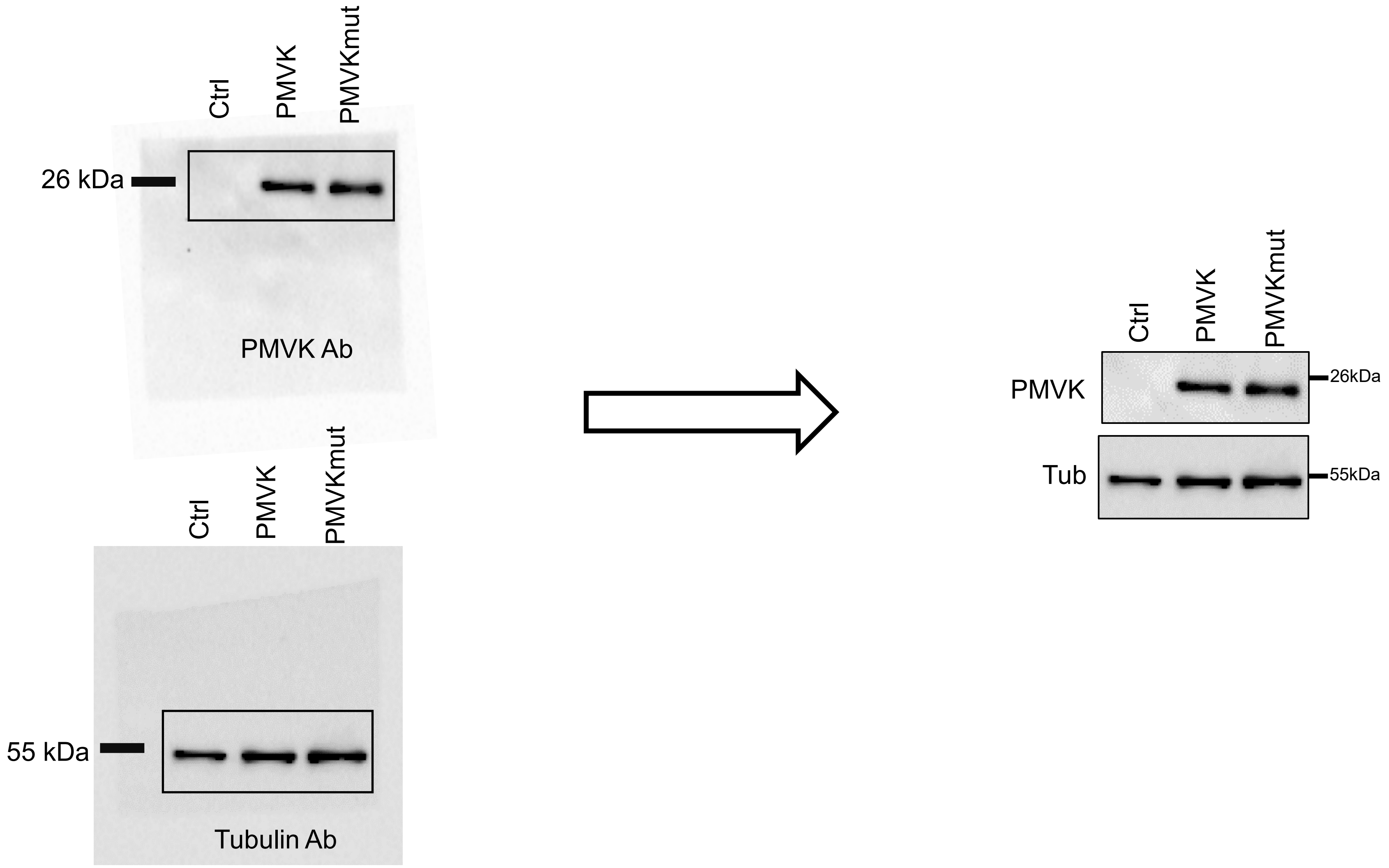

To generate Supplemental Figure 1B – upper panel

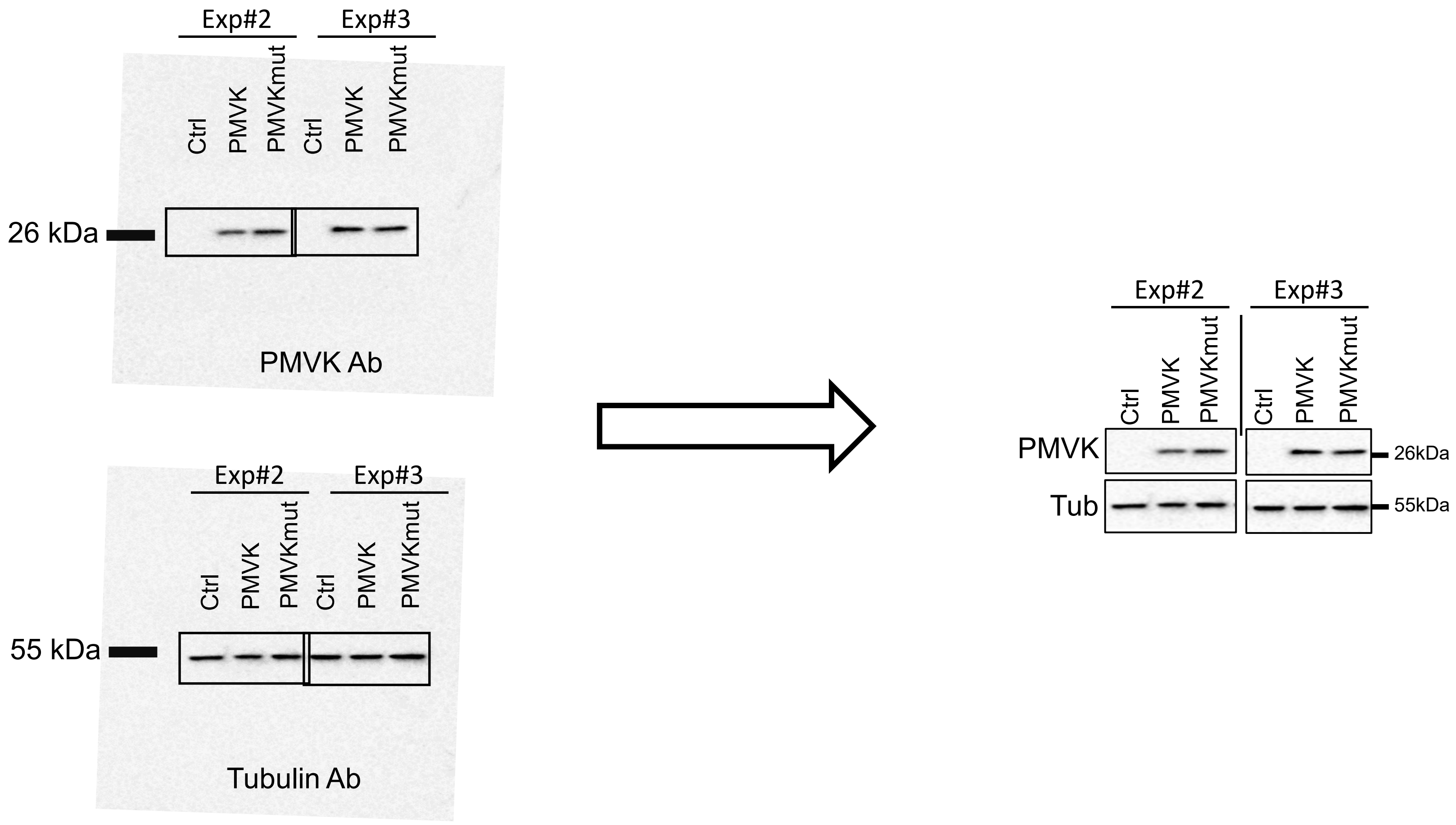

Supplemental materials, Ziegler et al

Uncropped Western blot

To generate Figure 1B – lower panel

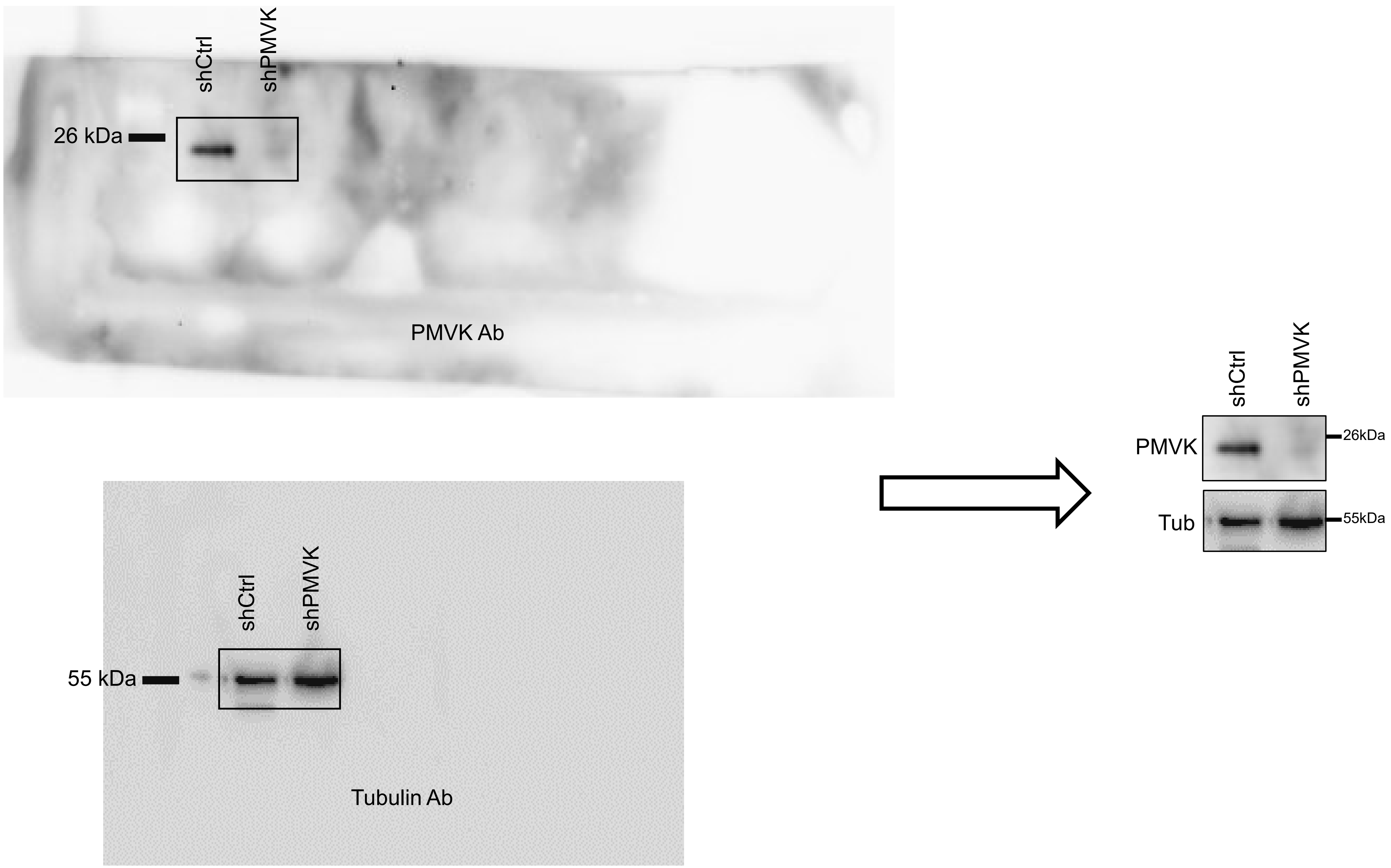

To generate Supplemental Figure 1B – lower panel

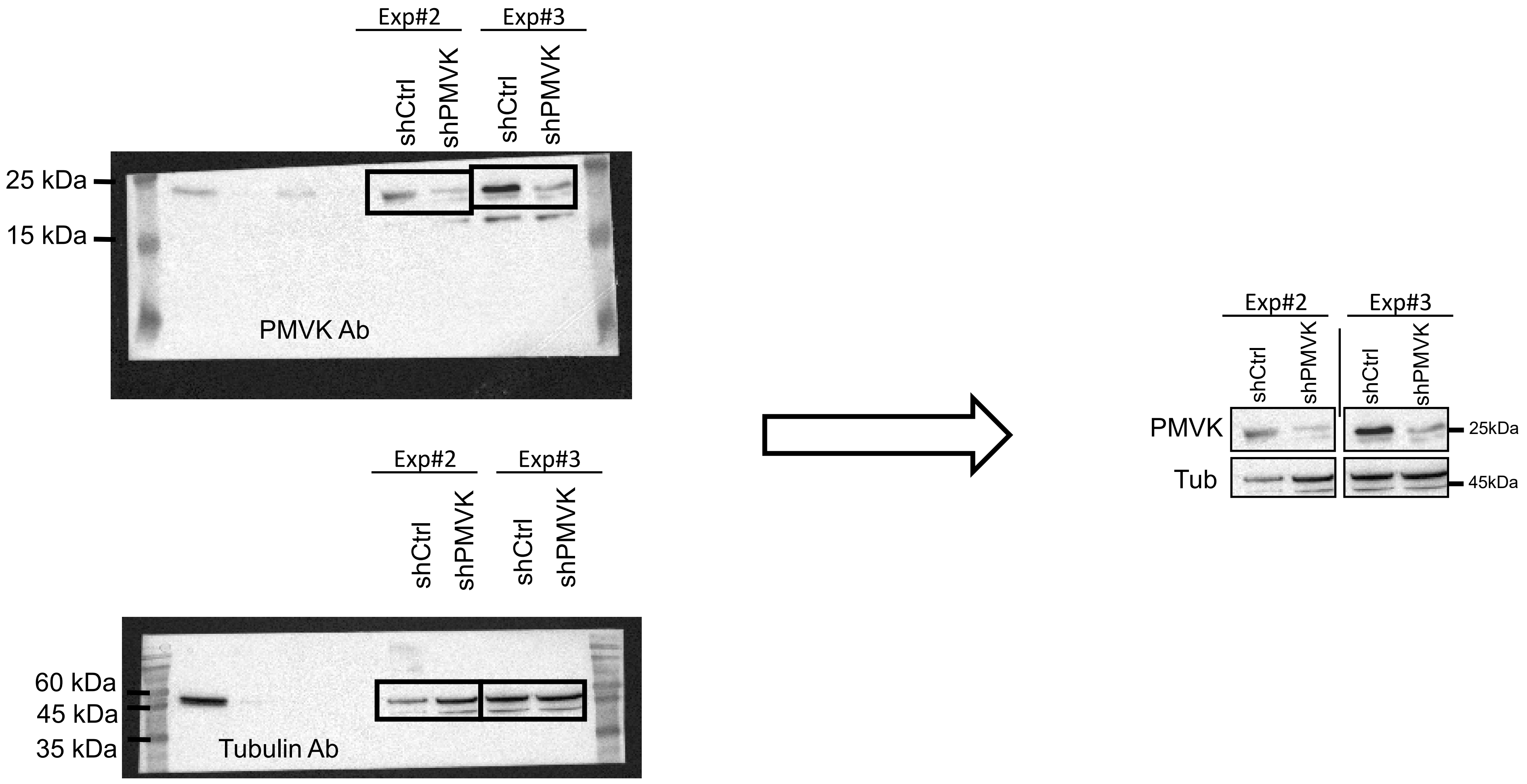

Supplemental materials, Ziegler et al

Uncropped Western blot

To generate Supplemental Figure 1H

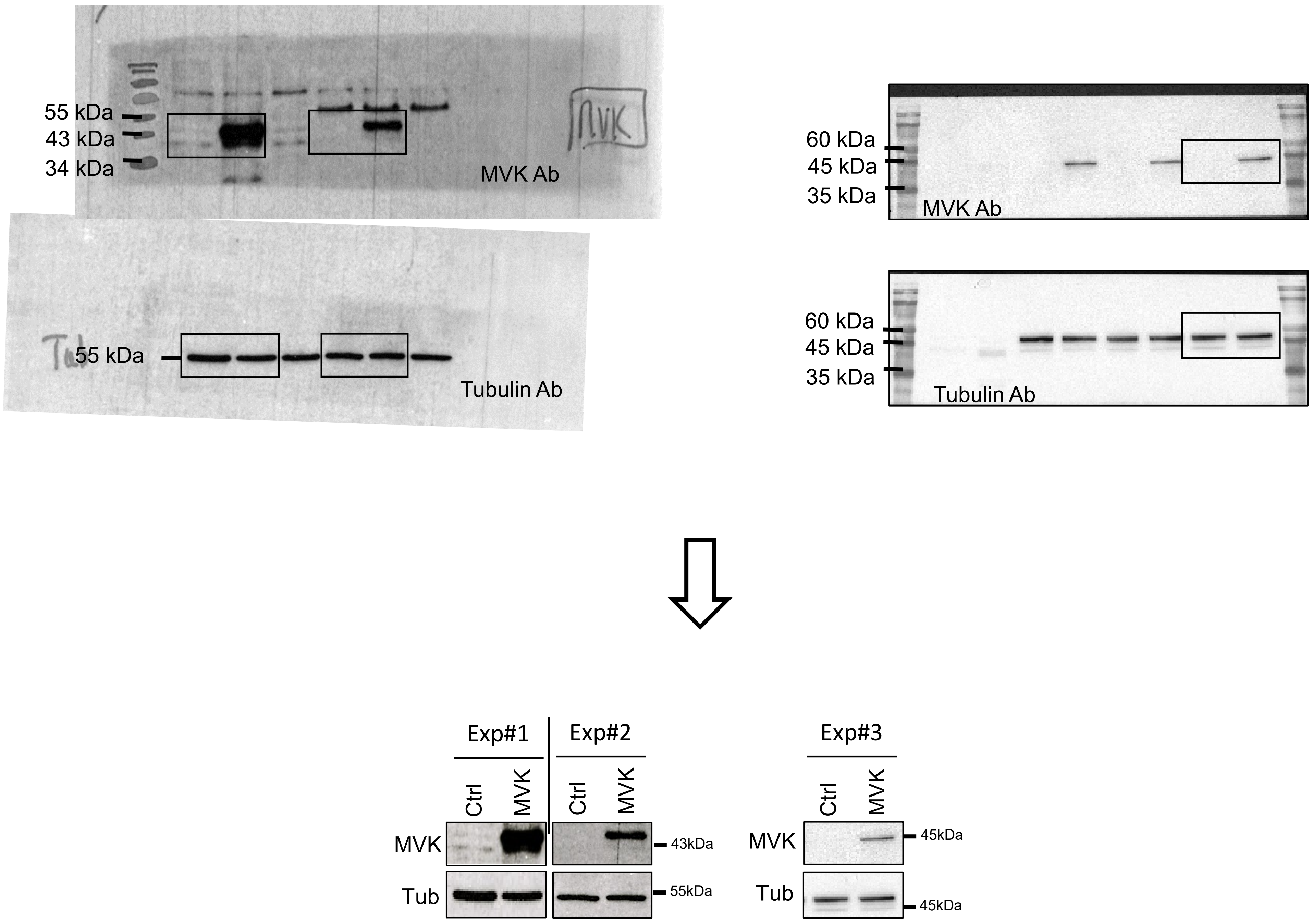

Supplemental materials, Ziegler et al

Uncropped Western blot

To generate Supplemental Figure 4I

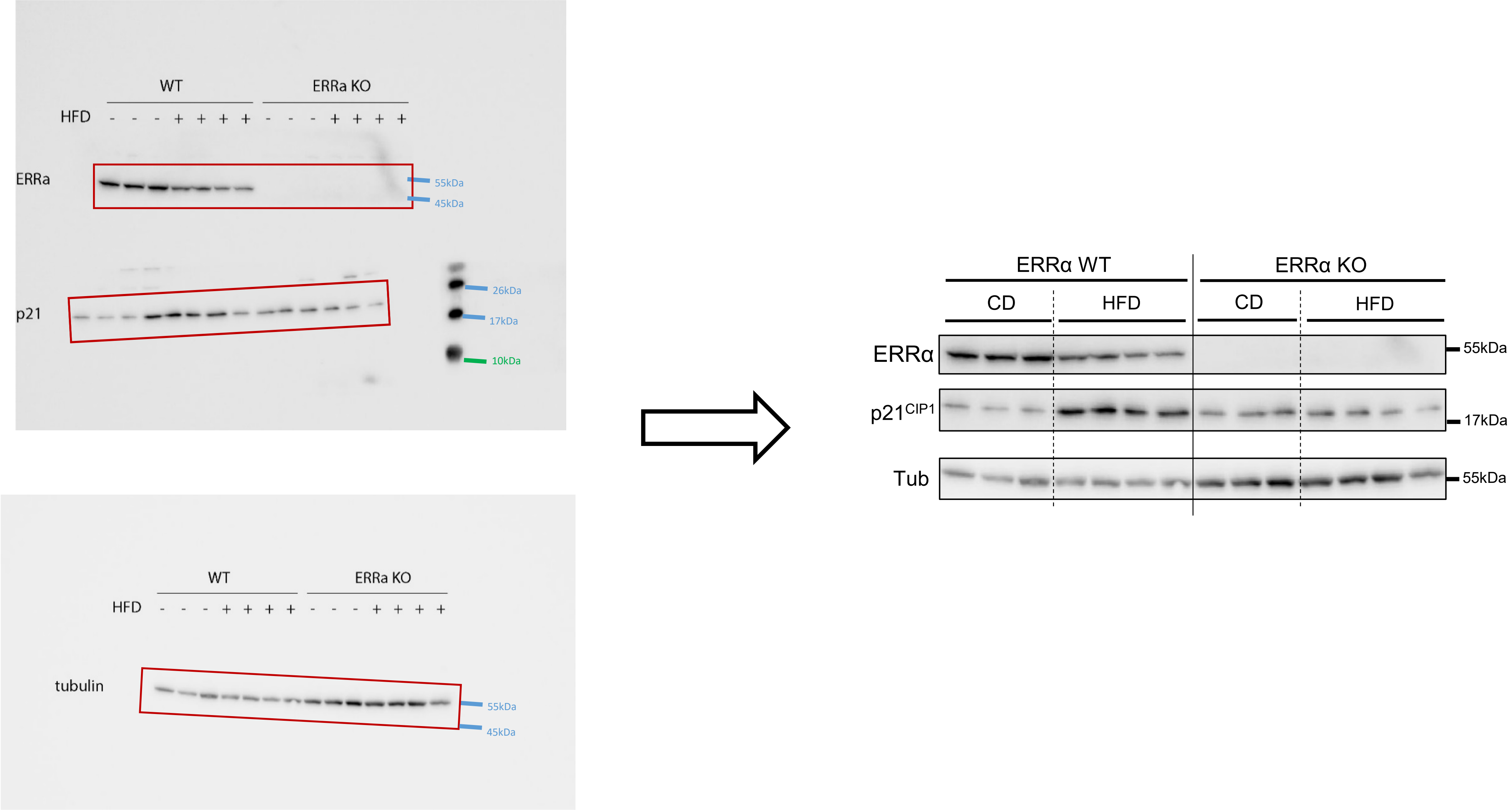

Supplement: Supplementary file 1 — Supplementary file [file 41514_2023_128_MOESM1_ESM.pdf]
