# Supplementary material for: Cholesterol biosynthetic pathway induces cellular senescence through ERRα
Source: NPJ Aging. 2024 Jan 12;10(1):5. doi: 10.1038/s41514-023-00128-y (PMC10786911; doi:10.1038/s41514-023-00128-y)
Supplement: Supplementary file 2 — Reporting Summary [file 41514_2023_128_MOESM2_ESM.pdf]

nature portfolio

Corresponding author(s): David BERNARD & Nadine MARTIN

Last updated by author(s): 2023-21-10

Reporting Summary

Nature Portfolio wishes to improve the reproducibility of the work that we publish. This form provides structure for consistency and transparency in reporting. For further information on Nature Portfolio policies, see our Editorial Policies and the Editorial Policy Checklist.

Statistics

For all statistical analyses, confirm that the following items are present in the figure legend, table legend, main text, or Methods section.

n/a	Confirmed	
☐	☒	The exact sample size (*n*) for each experimental group/condition, given as a discrete number and unit of measurement
☐	☒	A statement on whether measurements were taken from distinct samples or whether the same sample was measured repeatedly
☐	☒	The statistical test(s) used AND whether they are one- or two-sided<br>*Only common tests should be described solely by name; describe more complex techniques in the Methods section.*
☐	☒	A description of all covariates tested
☐	☒	A description of any assumptions or corrections, such as tests of normality and adjustment for multiple comparisons
☐	☒	A full description of the statistical parameters including central tendency (e.g. means) or other basic estimates (e.g. regression coefficient) AND variation (e.g. standard deviation) or associated estimates of uncertainty (e.g. confidence intervals)
☐	☒	For null hypothesis testing, the test statistic (e.g. *F*, *t*, *r*) with confidence intervals, effect sizes, degrees of freedom and *P* value noted<br>*Give P values as exact values whenever suitable.*
☒	☐	For Bayesian analysis, information on the choice of priors and Markov chain Monte Carlo settings
☐	☒	For hierarchical and complex designs, identification of the appropriate level for tests and full reporting of outcomes
☒	☐	Estimates of effect sizes (e.g. Cohen's *d*, Pearson's *r*), indicating how they were calculated

Our web collection on statistics for biologists contains articles on many of the points above.

Software and code

Policy information about availability of computer code

Data collection	N/A
Data analysis	N/A

For manuscripts utilizing custom algorithms or software that are central to the research but not yet described in published literature, software must be made available to editors and reviewers. We strongly encourage code deposition in a community repository (e.g. GitHub). See the Nature Portfolio guidelines for submitting code & software for further information.

Data

Policy information about availability of data

All manuscripts must include a data availability statement. This statement should provide the following information, where applicable:

- Accession codes, unique identifiers, or web links for publicly available datasets
- A description of any restrictions on data availability
- For clinical datasets or third party data, please ensure that the statement adheres to our policy

Data availability statement is provided.

Research involving human participants, their data, or biological material

Policy information about studies with human participants or human data. See also policy information about sex, gender (identity/presentation), and sexual orientation and race, ethnicity and racism.

Reporting on sex and gender	N/A
Reporting on race, ethnicity, or other socially relevant groupings	N/A
Population characteristics	N/A
Recruitment	N/A
Ethics oversight	N/A

Note that full information on the approval of the study protocol must also be provided in the manuscript.

Field-specific reporting

Please select the one below that is the best fit for your research. If you are not sure, read the appropriate sections before making your selection.

☒ Life sciences ☐ Behavioural & social sciences ☐ Ecological, evolutionary & environmental sciences

For a reference copy of the document with all sections, see nature.com/documents/nr-reporting-summary-flat.pdf

Life sciences study design

All studies must disclose on these points even when the disclosure is negative.

Sample size	Sample sizes for each experiment are indicated in the legends
Data exclusions	N/A
Replication	Experimental design was based on the use of at least 3 biological replicates as described in the figure legends.
Randomization	Mice from each genetic model were age- and sex-matched.
Blinding	N/A

Reporting for specific materials, systems and methods

We require information from authors about some types of materials, experimental systems and methods used in many studies. Here, indicate whether each material, system or method listed is relevant to your study. If you are not sure if a list item applies to your research, read the appropriate section before selecting a response.

Materials & experimental systems

n/a	Involved in the study	
☐	☒	Antibodies
☐	☒	Eukaryotic cell lines
☒	☐	Palaeontology and archaeology
☐	☒	Animals and other organisms
☒	☐	Clinical data
☒	☐	Dual use research of concern
☒	☐	Plants

Methods

n/a	Involved in the study	
☒	☐	ChIP-seq
☒	☐	Flow cytometry
☒	☐	MRI-based neuroimaging

Antibodies

Antibodies used	All antibodies references are provided in the supplemental Table S2 and/or in the method section.
Validation	PMVK, MVK and ERRa antibodies were validated as they recognize a protein at the expected size and as their level of expression is

Validation	decreasing after their knockdown (MVK and PMVK) or knockout (ERRa) and/or is increasing after their overexpression (PMVK). The other antibodies used were previously validated in different articles and by the providers (gH2AX, Tub and p21).

Eukaryotic cell lines

Policy information about cell lines and Sex and Gender in Research

Cell line source(s)	MRC5 : ATCC / 293 GP Cells : Clontech
Authentication	Cells were amplified and frozen upon receipt from the company before use.
Mycoplasma contamination	All cell lines used were tested negative for mycoplasma presence.
Commonly misidentified lines (See ICLAC register)	N/A

Animals and other research organisms

Policy information about studies involving animals; ARRIVE guidelines recommended for reporting animal research, and Sex and Gender in Research

Laboratory animals	Male ERRα KO (PMID:14585956) and WT controls in a C57BL/6J genetic background were used.
Wild animals	N/A
Reporting on sex	N/A
Field-collected samples	N/A
Ethics oversight	All animal experimentations were conducted in accord with accepted standards of humane animal care and all protocols were approved by the McGill Facility Animal Care Committee and the Canadian Council on Animal Care.

Note that full information on the approval of the study protocol must also be provided in the manuscript.
